# HKT1;5 Transporter Gene Expression and Association of Amino Acid Substitutions With Salt Tolerance Across Rice Genotypes

**DOI:** 10.3389/fpls.2019.01420

**Published:** 2019-11-04

**Authors:** Mohammad Umer Sharif Shohan, Souvik Sinha, Fahmida Habib Nabila, Shubhra Ghosh Dastidar, Zeba I. Seraj

**Affiliations:** ^1^Plant Biotechnology Laboratory, Department of Biochemistry and Molecular Biology, University of Dhaka, Dhaka, Bangladesh; ^2^Division of Bioinformatics, Bose Institute, Kolkata, India

**Keywords:** salt sensitive, salt tolerant, HKT1;5, gene expression, amino acid substitution, molecular dynamics simulation, Na^+^/K^+^ ratio

## Abstract

Plants need to maintain a low Na^+^/K^+^ ratio for their survival and growth when there is high sodium concentration in soil. Under these circumstances, the high affinity K^+^ transporter (HKT) and its homologs are known to perform a critical role with HKT1;5 as a major player in maintaining Na^+^ concentration. Preferential expression of HKT1;5 in roots compared to shoots was observed in rice and rice-like genotypes from real time PCR, microarray, and RNAseq experiments and data. Its expression trend was generally higher under increasing salt stress in sensitive IR29, tolerant Pokkali, both glycophytes; as well as the distant wild rice halophyte, *Porteresia coarctata*, indicative of its importance during salt stress. These results were supported by a low Na^+^/K^+^ ratio in Pokkali, but a much lower one in *P. coarctata*. HKT1;5 has functional variability among salt sensitive and tolerant varieties and multiple sequence alignment of sequences of HKT1;5 from *Oryza* species and *P. coarctata* showed 4 major amino acid substitutions (140 P/A/T/I, 184 H/R, D332H, V395L), with similarity amongst the tolerant genotypes and the halophyte but in variance with sensitive ones. The best predicted 3D structure of HKT1;5 was generated using Ktrab potassium transporter as template. Among the four substitutions, conserved presence of aspartate (332) and valine (395) in opposite faces of the membrane along the Na^+^/K^+^ channel was observed only for the tolerant and halophytic genotypes. A model based on above, as well as molecular dynamics simulation study showed that valine is unable to generate strong hydrophobic network with its surroundings in comparison to leucine due to reduced side chain length. The resultant alteration in pore rigidity increases the likelihood of Na^+^ transport from xylem sap to parenchyma and further to soil. The model also proposes that the presence of aspartate at the 332 position possibly leads to frequent polar interactions with the extracellular loop polar residues which may shift the loop away from the opening of the constriction at the pore and therefore permit easy efflux of the Na^+^. These two substitutions of the HKT1;5 transporter probably help tolerant varieties maintain better Na^+^/K^+^ ratio for survival under salt stress.

## Introduction

It has been predicted that food production will need to rise by 50% in the next 30 years to meet the demand of the growing population (Tian et al., 2016; Hunter et al., 2017). Since all cultivable lands are already in use, the only alternative is either to enhance productivity in existing land or to extend crop growth to marginal ones, such as those affected by salinity (Shahid and Al-Shankiti, 2013; Arzani and Ashraf, 2016; Dhankher and Foyer, 2018). Major food crop, i.e., rice, widely consumed in developing countries, is however sensitive to salinity, which is a major drawback for enhancing food production (Acosta-Motos et al., 2017).

Salinity stress harms plant yield mainly by osmotic stress and ion toxicity (Munns and Tester, 2008). During normal conditions, roots have lower water potential than the outside environment leading to an influx of water through channels known as aquaporin (Katsuhara et al., 2008; Chaumont and Tyerman, 2014). But during salt stress, soil water potential is reduced incapacitating the root’s ability to uptake water and causes water deficit (Pardo, 2010; Roy et al., 2014). The water deficiency signal is immediately transferred from root to shoot with a consequential reduction of turgor pressure and cell growth (Munns, 2005; Munns and Tester, 2008). This transmitted signal also lowers stomatal conductance and reduces biomass production and yield by promoting abscisic acid (ABA) formation (Munns, 2005; Munns and Tester, 2008; Roy et al., 2014). Over time, deficiency in water compounded by the influx of Na^+^ leads to ionic stress and toxicity. The term ion toxicity is used to refer to the impairment of cellular processes as a consequence of increased ion concentration, which is mainly due to excessive Na^+^ ions. Ion toxicity leads to inhibition of vital enzymatic reactions, photosynthesis, and protein synthesis (Yamaguchi et al., 2013; Hasanuzzaman et al., 2018). Besides photosynthetic processes are directly linked to biomass production and cellular reactions and therefore need to be protected from Na^+^ toxicity. Potassium (K^+^) is an essential macronutrient, similar to Na^+^ in terms of physiochemical properties (i.e., ionic radius and ion hydration energy). Therefore Na^+^ competes with K^+^ for many key enzymatic reactions. This competition for different enzymes that are activated by K^+^ results in disruption of cellular processes in roots and leaves (Almeida et al., 2017).

Protection mechanisms of plants against salinity stress have been elucidated by recent molecular and genetic studies (Al-Tamimi et al., 2016; Hussain et al., 2018; Yang and Guo, 2018; Akrami and Arzani, 2019). It has been suggested that maintenance of a high K^+^/Na^+^ or low Na^+^/K^+^ ratio is essential for the survival of plants under salt stress emphasizing on the regulation of Na^+^ transporters, water channels and signaling molecules in salt tolerance (Hilker and Schmülling, 2019; Wang et al., 2019). Many classes of Na^+^ transporters have been shown to play essential roles in Na^+^ homeostasis during salinity stress. Several classes of the transporters, NHX (sodium-hydrogen antiporter), HKT (high-affinity potassium transporter), CHX (cation-hydrogen exchanger), SOS1 (salt overly sensitive 1), and NSCC (non-selective cation channel) have shown significant involvement in Na^+^ transport through its sequestration in vacuoles (NHX, CHX), extrusion from cell circulation and recirculation (HKT), exclusion from root to soil (SOS1), and inclusion of Ca^2+^ (NSCC) to initiate signaling to compensate for the sodium load under saline conditions (Arzani and Ashraf, 2016; Quan et al., 2018; Yang and Guo, 2018; Arabbeigi et al., 2019; Bernstein, 2019). Improvement in salinity tolerance of crop plants has also been attributed to overexpression of some Na^+^ transporter genes (Liang et al., 2018).

The HKT family proteins are likely to be crucial during the salt stress tolerance response in plants (Munns and Tester, 2008; Roy et al., 2014; Ali et al., 2019). The first ever *HKT* gene found in a plant was *TaHKT2;1* (earlier known as *HKT1*) gene from wheat (Schachtman and Schroeder, 1994). TaHKT2:1 functions in high affinity Na^+^–K^+^ co-transport but shows Na^+^ selectivity in presence of a millimolar [Na^+^] in *Xenopus laevis* oocytes and yeast (Genet et al., 1995). Although some plants (dicots) such as *Arabidopsis thaliana* have only one *HKT* gene, referred to as *AtHKT1;1* (earlier known as *AtHKT1*), many plants (e.g., monocots) have multiple *HKT* genes (Ali et al., 2019).

The HKT family has several subclasses which exhibit a diversity of functions (Munns and Tester, 2008; Almeida et al., 2013; Roy et al., 2014). Based on the consensus reached in 2006, HKT has been classified into two groups depending on their transport characteristics and variable amino acid sequence with respect to the first pore domain (Platten et al., 2006). Members of the class 1 family have “selectivity filter” motif of Ser-Gly-Gly-Gly whereas class 2 have Gly-Gly-Gly-Gly (Mäser et al., 2002). The positioning of serine or glycine has crucial importance in the conductance ability. The presence of serine facilitates Na^+^ transport over other cations, while the presence of glycine allows transport of both Na^+^ and K^+^ depending on the external concentration of ions (Platten et al., 2006; Kronzucker and Britto, 2011). There are some notable exceptions to this rule which is observed in *Oryza sativa* OsHKT2;1, *Eucalyptus camaldulensis* EcHKT1;2, and *Thellungiella salsuginea* TsHKT1;2. Though OsHKT2;1 has Ser-Gly-Gly-Gly selectivity filter motif, it is defined as class 2 HKT family protein (Mäser et al., 2002; Haro et al., 2005). EcHKT1;2 transports both Na^+^ and K^+^ which is normally observed in case of class 2 HKT protein (Gassmann et al., 1996; Platten et al., 2006). TsHKT1;2 is also an exception as it is a two-way transporter moving K^+^ and Na^+^ in opposite directions (Mäser et al., 2002; Ali et al., 2016). Studies conducted on wheat showed that transport properties of TmHKT1;5 and TaHKT1;5 provide improved Na^+^ exclusion leading to improved salinity tolerance (Xu et al., 2018). This indicates that determination of substrate selectivity is not dependent on selectivity filter alone and other structural elements may also be involved.

**Figure 1:**
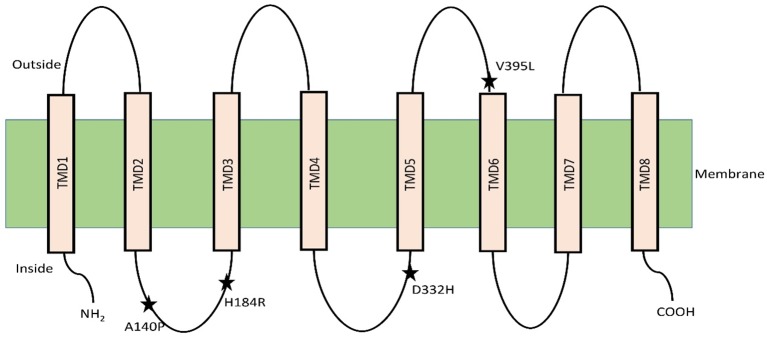
OsHKT1;5 model with eight transmembrane domain based on hydrophobicity plot analysis. Residue substitutions between Nona Bokra and Koshihikari are indicated by asterisks. Diagram adapted from (Ren et al., 2005).

The selective presence of variants of the class 1 HKT family protein in salt sensitive and tolerant varieties has led to hypotheses regarding the mechanism of plant protection against excess Na^+^ level (Gong et al., 2005; Wu et al., 2012; Vera-Estrella et al., 2014). The discovery of quantitative trait loci for the class 1 HKT transporter have shown how the accumulation of Na^+^ in leaves of wheat and rice is controlled in monocotyledonous plants (Mishra et al., 2016; Zhang et al., 2018). In rice, a specific variant of the transporter SKC1 was characterized and identified to be involved in maintaining shoot K^+^ concentration under NaCl stress in salt tolerant (Nona Bokra), but not in the salt sensitive (Koshihikari) variant. This SKC1 was found to match the amino acid sequence of OsHKT1;5 through homology search with existing database sequences (Ren et al., 2005). In *Aegilops cylindrica*, the *AecHKT1;5* was found to be involved in shoot to root transport, combined with exclusion of excessive Na^+^ from the root (Arabbeigi et al., 2018). Mapping of HKT1;5 gene in barley using genome-wide association study approach provided evidence of the function in unloading of Na^+^ from xylem and thus controlling distribution in the shoots (Hazzouri et al., 2018). The rice OsHKT1;5 has been hypothesized to control Na^+^ flow in the recirculation process from xylem vessel into xylem parenchyma thereby facilitating shoot K^+^ homeostasis by maintaining the Na^+^/K^+^ ratio (Ren et al., 2005; Platten et al., 2013). Normal and heterologous expression analysis of the tolerant variant of the gene in *Xenopus* oocytes and Na^+^ and K^+^ accumulation in roots and shoots has confirmed this role of OsHKT1;5 in rice. Comparison between nucleotide differences of HKT1;5 of the salt tolerant variety Nona Bokra and sensitive variety Koshihikari has shown that four vital amino acid substitutions regulate the Na^+^ transport efficiency of HKT1;5. The authors did not however propose specific roles for the substituted amino acids (140 P/A/T/I, 184 H/R, D332H, V395L) (Ren et al., 2005). The topological study indicated that OsHKT1;5 has eight transmembrane domains and the amino acids variations lie in the loop regions between the domains. Two of these variations are in the loop between TMD2 and TMD3. The third one is in the loop between TMD4 and TMD5 and the last one is between TMD5 and TMD6 (**Figure 1**) (Ren et al., 2005).

AtHKT1;1 and OsHKT1;5 which are expressed in xylem parenchyma cells maintain the flow of Na^+^ and K^+^ in opposite directions in the xylem sap (Horie et al., 2005; Ren et al., 2005). One hypothesis suggests that transport of Na^+^ by HKT1;5 into xylem parenchyma causes depolarization-activation of K^+^ channel (SKOR, shaker type outward-rectifying K^+^ channel and NOR, nonselective outward-rectifying channel) resulting in transportation of K^+^ into xylem sap (Horie et al., 2009). The functional mechanism of HKT1;5 during salt stress is still a matter of debate due to some exceptions in transport activity of class 1 HKT transporters as seen in TsHKT1;2, and EsHKT1;2 (Gassmann et al., 1996; Ali et al., 2012). Another deviation was reported for the halophyte *T. salsuginea* TsHKT1;2 protein (HKT1), which is a two way transporter, and shows the ability to transport K^+^ along with Na^+^ in opposite directions (Mäser et al., 2002; Ali et al., 2016). In the present study, we checked the gene expression pattern of HKT1;5 in IR29, Pokkali, and *P. coarctata* as well as from seven microarray-based gene expression studies. Further, we collected the sequences of *HKT1;5* from some accessions of *O. sativa* and their relatives, *Oryza brachyantha*, *Oryza rufipogon, Oryza glaberrima, Oryza nivara* as well as the wild halophytic rice *P. coarctata* (or the older nomenclature, *Oryza coarctata)*, and searched for amino acid substitutions in genotypes known to be salt tolerant *versus* salt sensitive ones. We observed that two amino acids aspartate (332) and valine (395) present across the plasma membrane were conserved across salt tolerant genotypes (including halophytes), which represented two of the positions identified by Ren et al. (2005). This led us to hypothesize a model involving two amino acid substitutions across the membrane in the transporter OsHKT1;5 for efficient transport of Na^+^. The model shows how these two amino acids can confer salt tolerance in halophytes as well as rice genotypes by maintenance of an efficient Na^+^/K^+^ ratio in shoots.

## Materials and Methods

### Plant Growth Conditions and Treatments

From Teknaf (21.0557° N, 92.2040° E) and Bakkhali River estuary (21.447340° N, 92.003142° E) of Cox’s Bazar District *P. coarctata* was collected. One-month-old young *P. coarctata* already established in soil (net house of Plant Biotechnology Laboratory, University of Dhaka) were gently removed from soil and the roots placed in netted styrofoam floated in a hydroponic solution after washing off the soil in tap water. For the establishment of *P. coarctata* in the hydroponic system and Yoshida culture solution (Flowers et al., 1990) were used. Meanwhile the sensitive variety IR29 and tolerant variety Pokkali seeds were germinated and plants were kept in hydroponic system (Platten et al., 2013).

The seedlings of IR29 and Pokkali were grown in the hydroponic solution for 3 weeks. The shoots with roots and rhizomes of *P. coarctata* required about a month for stabilization and attained a height of around 10–15 cm (almost the same size as the seedlings of IR29 and Pokkali) in the hydroponic solution. The rice and *P. coarctata* plants were then subjected to salt stress (NaCl) at daily increment of 50 mM until day 2 (when it reached 100 mM) and day 4 (when it reached 200 mM). So, the tissue collected for RNA isolation were on 3^rd^ day, 24 h after the application of the last 50 mM increment for 100 mM salt stress plants and on 5^th^ day, 24 h after the application of the last 50 mM increment for 200 mM salt stress. The control plants were subjected to hydroponics without salt stress and tissue were collected on 3^rd^ day for RNA isolation. For each condition, there were six biological replicates. The control plants were also used to check the tissue specific expression.

### Ribonucleic Acid Extraction and Complementary DNA Synthesis

The roots and shoots of the seedlings (salt and controls) were harvested directly into liquid nitrogen for total RNA extraction. Total RNA was extracted from the shoots and roots using the TRIzol reagent (Ambion, Invitrogen) following the manufacturer’s protocol. cDNAs were synthesized from 1 µg total RNA (pre-treated by DNase I, Roche) of transgenic and non-transgenic root and shoot according to the Invitrogen two-step reverse-transcription (RT)-PCR manufacturer’s protocol.

### Real-Time Quantitative Polymerase Chain Reaction Analysis

Quantitative real-time PCR (qPCR) was performed in a 10 µl reaction using SYBR Green (Bio‐Rad, USA) with gene-specific internal primers pairs (**Supplementary Table 1**) in a CFX96 TM Real‐Time PCR Detection System (Bio‐Rad, USA). PCR efficiency (90–95%) was verified and amplification specificity was validated by melt curve analysis at the end of PCR cycle. All reactions were performed with six biological and three technical replicates. The relative expression levels of HKT1;5 gene from different plants were calculated using the Pfaffl formula (ratio = 2^-ΔΔCt^) method with elongation factor‐α (EF‐α) used as the normalization control. Here ΔΔCt = (ΔCt sample - ΔCt control); ΔCt sample = (ΔCt target - ΔCt ref) for all sampling times and NaCl concentrations; and ΔCt control = (ΔCt target - ΔCt ref) (Pfaffl, 2001).

### *In Silico* Gene Expression Analysis

In current study, we have analyzed expression of HKT1;5 in different part of rice across biotic and abiotic stress based on mRNAseq (**Supplementary Table 2**) and microarray data (**Supplementary Table 3**) collected from Genevestigator (https://genevestigator.com/gv/). Moreover, we used this tool to analyze the relative expression of HKT1;5 gene under different salt stress conditions in rice (Hruz et al., 2008) (**Supplementary Table 4**).

### Measurement of Na^+^, K^+^ Content

For the measurement of sodium and potassium concentrations in shoot and root, three biological replicates were collected at 0, 100, and 200 mM for each salt stress conditions, plants were washed in flowing tap water for 30 s, and the oven-dried plants from each biological replicate were ground and analyzed by a Flame Photometer 410 (Sherwood, UK) after 48 h of extraction with 0.05N HCl following the procedure described by Yoshida et al. (1971). At first the standard curve was plotted for both sodium and potassium using 10, 8, 6, 4, 2, 1, and 0.5 ppm sodium and potassium standard solutions. Machine values for the samples were plotted on the standard curve to get the ppm values of sodium and potassium content. Concentrations were calculated as percent of dry weight following the same calculation method used by Amin et al. (2016).

mmol per gram dry mass=(ppm value*dilution factor)/(equivalent weight*1,000*dry weight in gram)

The Na^+^/K^+^ ratio was measured dividing the sodium content by the potassium content. All statistical analyses were done using R software packages.

### Statistical Analysis

The effect of variables of gene expression between salt concentrations were tested using the one-way analysis of variance (ANOVA) followed by mean comparisons through Tukey’s HSD *post hoc* test. P-values of p < 0.05 and p < 0.01 respectively were considered as significant and highly significant change against controls. Values are expressed as mean ± SEM (standard error of mean). The reproductive screening data were compared between all groups using a one-way analysis of variance (ANOVA), with a *post hoc* Tukey HSD analysis used for multiple pairwise comparisons. The software R was used for all analysis.

### Retrieval of HKT1;5 Sequences and Finding Open Reading Frame of Nucleotide Sequence and Translating to Protein Sequence

HKT1;5 transporter sequences of salt tolerant and salt sensitive *Oryza* varieties were obtained by searching using the keyword “HKT1;5 and *Oryza*” from National Center for Biotechnology Information (NCBI) database (ncbi.nlm.nih.gov/). These are shown in **Table 1**. The Sequence Manipulation Suite (http://www.ualberta.ca/~stothard/javascript/) is a collection of freely available JavaScript applications for molecular biologists. ORF Finder looks for open reading frames (ORFs) in provided DNA sequences and returns ORFs along with protein translation (Stothard, 2000). We also collected 137 rice varieties and sequence accessions from 3,000 rice genome project and checked their tolerance ability under 120 mM salt stress for 2 weeks (Li et al., 2014).

**Table 1:** Retrieval of protein and nucleotide sequences of HKT1;5 from different salt sensitive and tolerant cultivars.

Protein sequence			
*Accession*	*Variety*	*Tolerant/sensitive*	*Reference*
*AFY08293.1*	*Oryza sativa* cv. Nona Bokra	Tolerant	(Platten et al., 2013)
*ABN48306.1*	*Oryza sativa* cv. Pokkali	Tolerant	(Platten et al., 2013)
*AFY08290.1*	*Oryza sativa* cv. Ta Lay	Tolerant	(Platten et al., 2013)
*AFY08295.1*	*Oryza sativa* cv. FL478	Tolerant	(Platten et al., 2013)
*AFY08294.1*	*Oryza sativa* cv. Hasawi	Tolerant	(Platten et al., 2013)
*AFY08297.1*	*Oryza sativa* cv. Agami	Tolerant	(Walia et al., 2007)
*AFY08296.1*	*Oryza sativa* cv. Basmati 217	Tolerant	(Platten et al., 2013)
*EEC70498.1*	*Oryza sativa* cv. 93-11	Sensitive	(Platten et al., 2013)
*ADM87303.1*	*Oryza sativa* IR29	Sensitive	(Platten et al., 2013)
*XP_015631953.1*	*Oryza sativa* cv. Nipponbare	Sensitive	(Platten et al., 2013)
*XP_015688361.1*	*Oryza brachyantha*	Unknown	
*AMY98961.1*	*Oryza coarctata*	Tolerant	(Bal and Dutt, 1986)
*AFY08292.1*	*Oryza glaberrima*	Tolerant	(Platten et al., 2013)
*AFY08287.1*	*Oryza rufipogon*	Tolerant	(Tian et al., 2011)
Nucleotide sequence			
*KT796051.1*	Oryza sativa isolate NPT11	Sensitive	(Khush, 1995; Peng et al., 1999)
*KT796050.1*	*Oryza sativa* isolate PB1	Sensitive	(Platten et al., 2013)
*KT796049.1*	*Oryza sativa* isolate CSR27	Tolerant	(Reddy et al., 2017)
*KT796048.1*	*Oryza sativa* isolate CSR11	Tolerant	(Tiwari et al., 2016)
*KT796047.1*	*Oryza sativa* isolate VSR156	Tolerant	(Senguttuvel et al., 2016)
*KT796046.1*	*Oryza sativa* isolate MI48	Moderately Tolerant	(Mishra et al., 2016)
*KT796044.1*	*Oryza sativa* isolate PUSA 44	Moderately Tolerant	(Mishra et al., 2016)
*KT796058.1*	*Oryza nivara* isolate 336684	Tolerant	(Mishra et al., 2016)
*KT796056.1*	*Oryza nivara* isolate NKSWR186	Tolerant	(Mishra et al., 2016)

### Multiple Sequence Alignment and Construction of Phylogenetic Tree

The retrieved sequences were subjected to multiple sequence alignment in MEGA7 software for locating any variant substitutions between salt sensitive and salt tolerant varieties. The CLUSTALW algorithm with default parameters was used to prepare the alignment (Kumar et al., 2016). For the analysis of evolutionary divergence, phylogenetic tree was constructed using maximum likelihood method in MEGA7 software where bootstrap value was set to 500 (Kumar et al., 2016).

### Subcellular Localization Prediction and Transmembrane Properties

Subcellular localization was predicted using consensus results of localization predictor; i) Plant-PLoc (version 2) http://www.csbio.sjtu.edu.cn/bioinf/plant/ (Chou and Shen, 2010), ii) CELLO (version 2.5) http://cello.life.nctu.edu.tw/ (Yu et al., 2014). The MEMPACK prediction server which is a component of PSIPRED portal, was used to find out the transmembrane topology (Nugent et al., 2011; Buchan and Jones, 2019).

### Homology Modeling and Structure Validation

Homology modeling of HKT1;5 transporter Nona Bokra (AFY08293.1) was performed using ExPASy server which is a web based environment for homology modeling (Gasteiger et al., 2003). Best six models (template ID: 3pjz.1.A, 6hra.1.A, 4j7c.1.I, 5but.1.E, 5mrw.1.A, 4j9u.1.A) were chosen based on identity and global model quality estimation (GMQE) coverage > 0.39 (Vieira-Pires et al., 2013). These models were further validated using ERRAT, Verify3D, PROCHECK, RAMPAGE (Colovos and Yeates, 1993; Laskowski et al., 1993; Eisenberg et al., 1997; Gelly et al., 2011). Ramachandran plot was made with the modeled structure using PROCHECK in order to validate the structure. The model was then submitted to the ProSA protein structure analysis tool to calculate z-score (Wiederstein and Sippl, 2007). The predicted structure was later superimposed on the template structure using Pymol for interactive view and calculation of RMSD (root mean square deviation) value (DeLano, 2002).

### System Preparation and Simulation Details

The structure of the modeled tolerant variety Nona Bokra was capped with acyl and amide groups at the N- and C-terminal end respectively to neutralize the charged terminals using Chemistry at Harvard Macromolecular Mechanics (CHARMM) modeling and simulation suite (Brooks et al., 2009). The structure was refined again by minimization and then it was packed in a bilayer of 100 POPC lipids in each leaflet using the strategy of Kandt et al. (2007). The bilayer was built using CHARMM-GUI membrane builder (Jo et al., 2009) and then further equilibrated for 20 ns. The protein membrane system was solvated in TIP3P water (Jorgensen et al., 1983) layers of height ~ 25 Å at both ends of the assembly along Z-axis. After initial neutralization using potassium and chloride ions, two separate systems were prepared. One in 0.15 M KCl solution (NBK-I) and one in mixed salt solution of 0.15 M KCl and 0.15 M NaCl (NBK-II) to mimic the salt stress condition. The structure of the sensitive variety, Nipponbare, was obtained by mutating residues in Nona Bokra using CHARMM scripts and minimized. The sequence of Nipponbare and Nona Bokra are same except for 140, 184, 332, and 395 amino acid positions (Nipponbare : XP_015631953.1, Nona Bokra: AFY08293.1). Similar to the Nona Bokra, two systems at different salt mixtures were also prepared for Nipponbare (NPB-I: 0.15 M KCl; NPB-II: 0.15 M KCl and 0.15 M NaCl).

CHARMM36 force field parameters (Best et al., 2012); (Klauda et al., 2010) were used to describe protein and lipid molecules and all simulations were run in NAMD 2.12 simulation engine (Phillips et al., 2005). All the systems were minimized first and then equilibrated for ~ 2 ns with an integration time step of 1 fs in several cycles of isothermal-isobaric (NPT) simulations. Initially larger harmonic restraints were applied on peptide (10 kcal mol^-1^ Å^-2^ on backbone atoms and 5 kcal mol^-1^ Å^-2^ on side chain atoms) and lipid head-group atoms (5 kcal mol^-1^ Å^-2^) which were further reduced in subsequent cycles to relax the systems. Further, all the systems were run for 60 ns each with a time step of 2 fs after constraining all H-containing bonds using the SHAKE algorithm (Ryckaert et al., 1977). Temperatures were fixed at 303 K for all the simulation using Langevin dynamics with a damping constant of 1 ps^-1^ and pressure were maintained at 1 atm using Langevin piston method (Feller et al., 1995). The periodic boundary condition was enforced in all the simulations considered for the present study. Short range non-bonded interactions were estimated for atoms with 12 Å and long-range electrostatic interactions were taken care of using particle mesh Ewald method (Darden et al., 1993) with a grid size of 1 Å. Altogether, the four systems were simulated for 240 ns.

## Results

### The Tissue-Specific Expressions of HKT1;5

**Figure 2:**
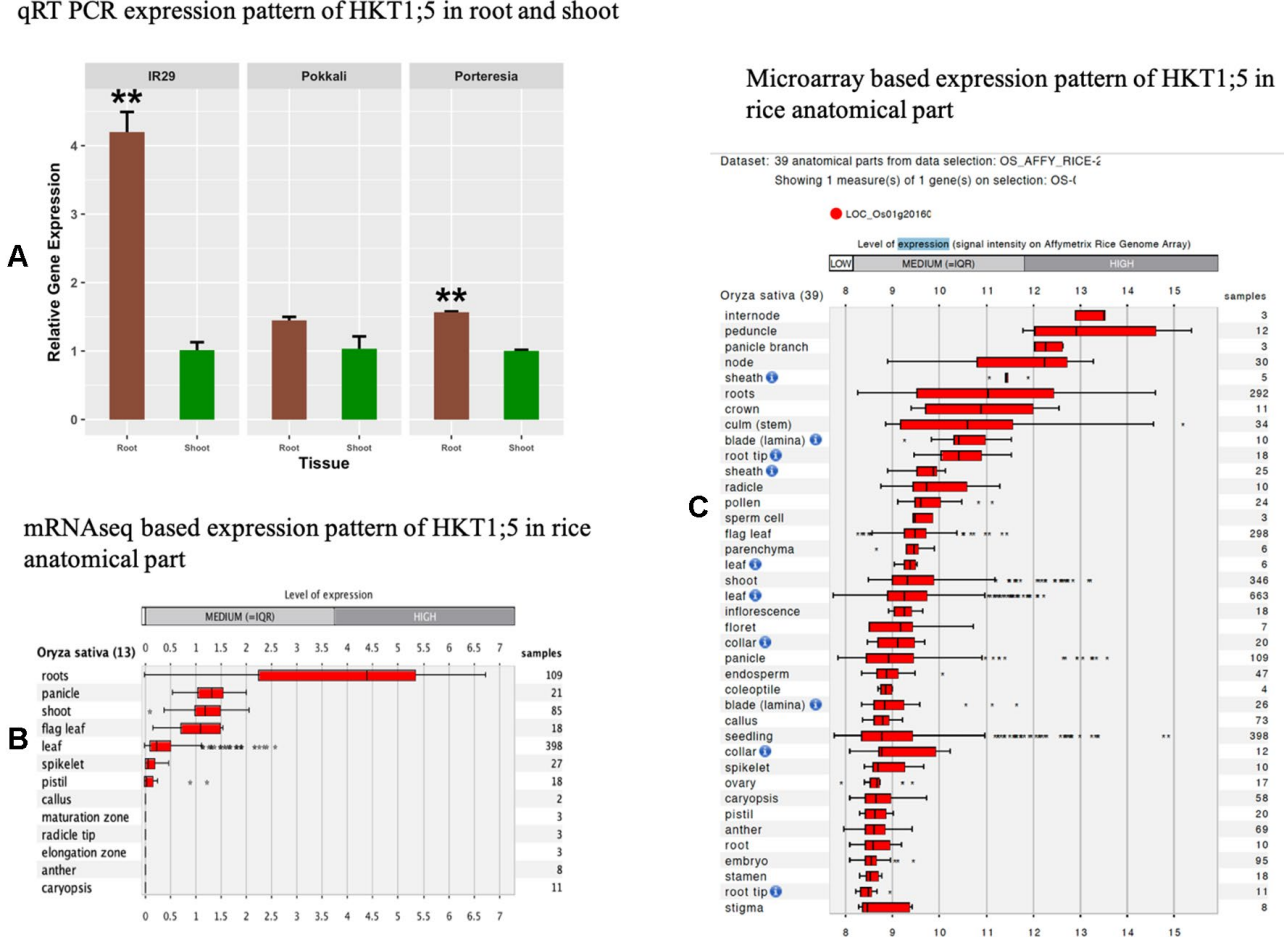
The relative expression levels of HKT1;5. **(A)** in roots and shoots of *Porteresia coarctata*, Pokkali, and IR29 under control condition (no additional NaCl). Elongation factor-α (EF-α) used as reference. Values are expressed as mean ± SEM (standard error of mean) and bars indicate SDs. * and ** denoted p 0.05 and p 0.01 respectively as compared to shoot. All expression analysis was performed with six biological and three technical replicates. Next, level of expression of HKT1;5 in different anatomical parts of rice based on **(B)** mRNAseq data (**Supplementary Table 2**) and **(C)** microarray based data (**Supplementary Table 3**) extracted from Genevestigator expression analysis tools (https://genevestigator.com/gv/) shows preferential expression of HKT1;5 in roots.

For investigating the trend of tissue specific gene expression of HKT1;5 in *P. coarctata*, Pokkali, and IR29, we performed qRT-PCR to test relative expression of HKT1;5 gene in shoots and roots of *P. coarctata*, Pokkali, and IR29 in control conditions without stress. The result showed the HKT1;5 is mainly expressed in roots compared to shoots as the expression is found to be lower in the latter (**Figure 2A**). This indicated the tissue specific positioning of HKT1;5 in roots. This result from the mRNAseq (**Figure 2B**) and microarray (**Figure 2C**) data analysis from Genevestigator software shows that expression of HKT1;5 is higher in roots compared to shoots based on the number of samples and expression level.

### The Expression Patterns of HKT1;5 in Roots Under Different Concentrations of Sodium Chloride

**Figure 3:**
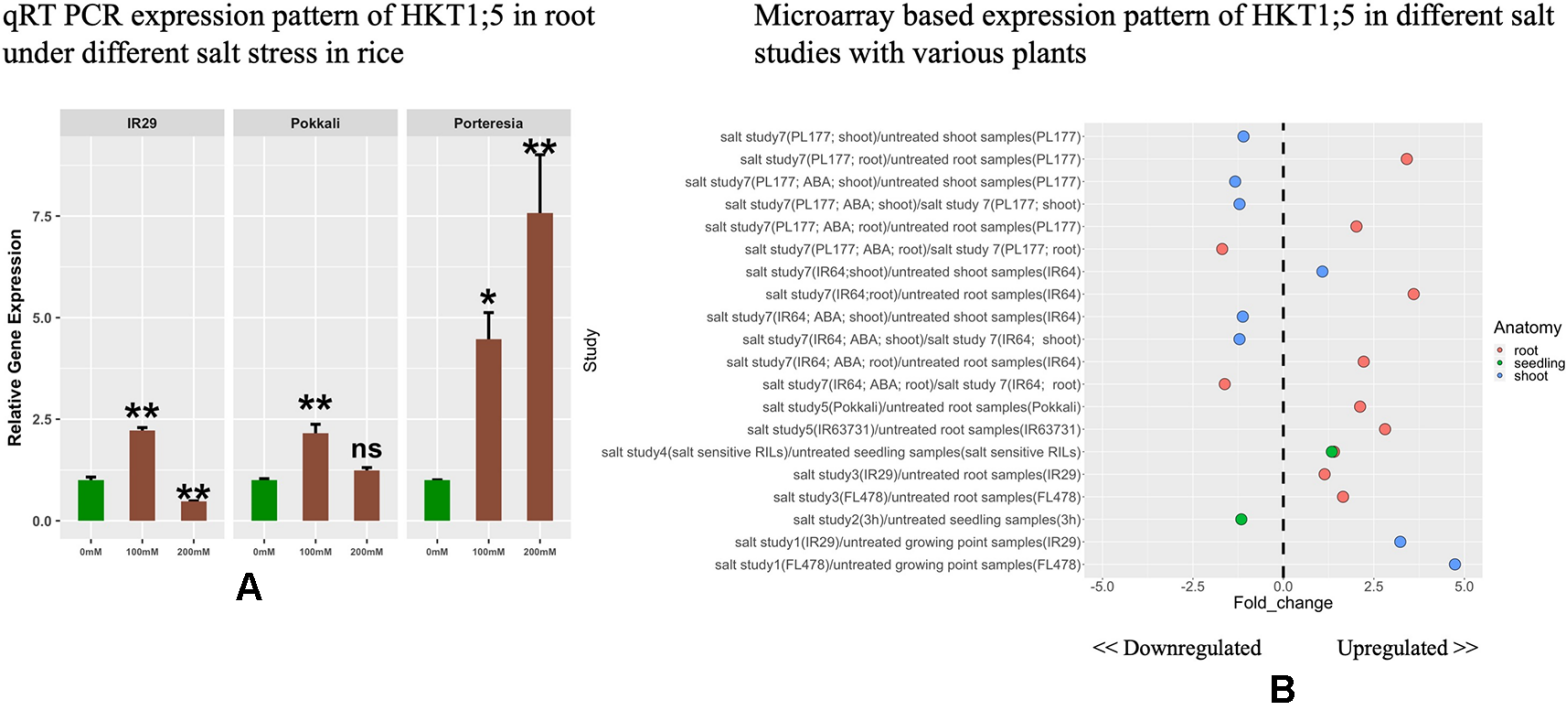
**(A)** The relative expression levels of HKT1;5 in IR29, Pokkali, and *P. coarctata* under 100 and 200 mM NaCl after 24 h. All expression analysis was performed with six biological and three technical replicates. Elongation factor-α (EF-α) used as internal reference. Experiments were repeated at least three times values are given as mean ± SEM.* and ** denoted p 0.05, p 0.01 and ns indicate no significance respectively as compared to control. **(B)** Different expression pattern of HKT1;5 extracted from microarray based data from Genevestigator expression analysis tools (https://genevestigator.com/gv/) (**Supplementary Table 4**).

The expressions of HKT1*;5* in roots were investigated under different concentrations of NaCl (**Figure 3A**). HKT1;5 was induced by 100 mM NaCl in a similar pattern for IR29 and Pokkali after 24 h. It was observed that the increase in expression of HKT1;5 was induced by around 2.4 fold under 100 mM NaCl in IR29 and Pokkali compared to control. Despite this similar induction of HKT1;5, IR29 is unable to survive in 100 mM salt stress for more than 2 weeks, whereas Pokkali can survive (Singh and Sarkar, 2014). At 200 mM NaCl, IR29 survives only 3–4 days, while Pokkali up to 10 days. At 24 h, the expression of IR29 was observed to drop whereas in Pokkali it continued to increase by 1.2 fold compared to control, providing an explanation of why 200 mM NaCl stress is lethal for IR29 survival and the plant enters a death cycle. The halophyte *P. coarctata* on the other hand can survive under salt stress up to 400 mM salt stress (Jagtap et al., 2006). Under 100 and 200 mM the expression of HKT1;5 in *P. coarctata* showed gradual increase and the value reached 7.5 fold increase under 200 mM stress compared to control. Microarray-based expression analysis based on 22 different conditions (**Supplementary Table 4**) revealed that HKT1;5 gets upregulated in roots under salt stress in different sensitive and tolerant varieties of rice and the expression is found to be downregulated in shoots (**Figure 3B**). This provides insights into the important role it plays during salt stress in roots of rice.

### Different Na^+^/K^+^ Ratio Maintenance at 24 h in Root and Shoot

**Figure 4:**
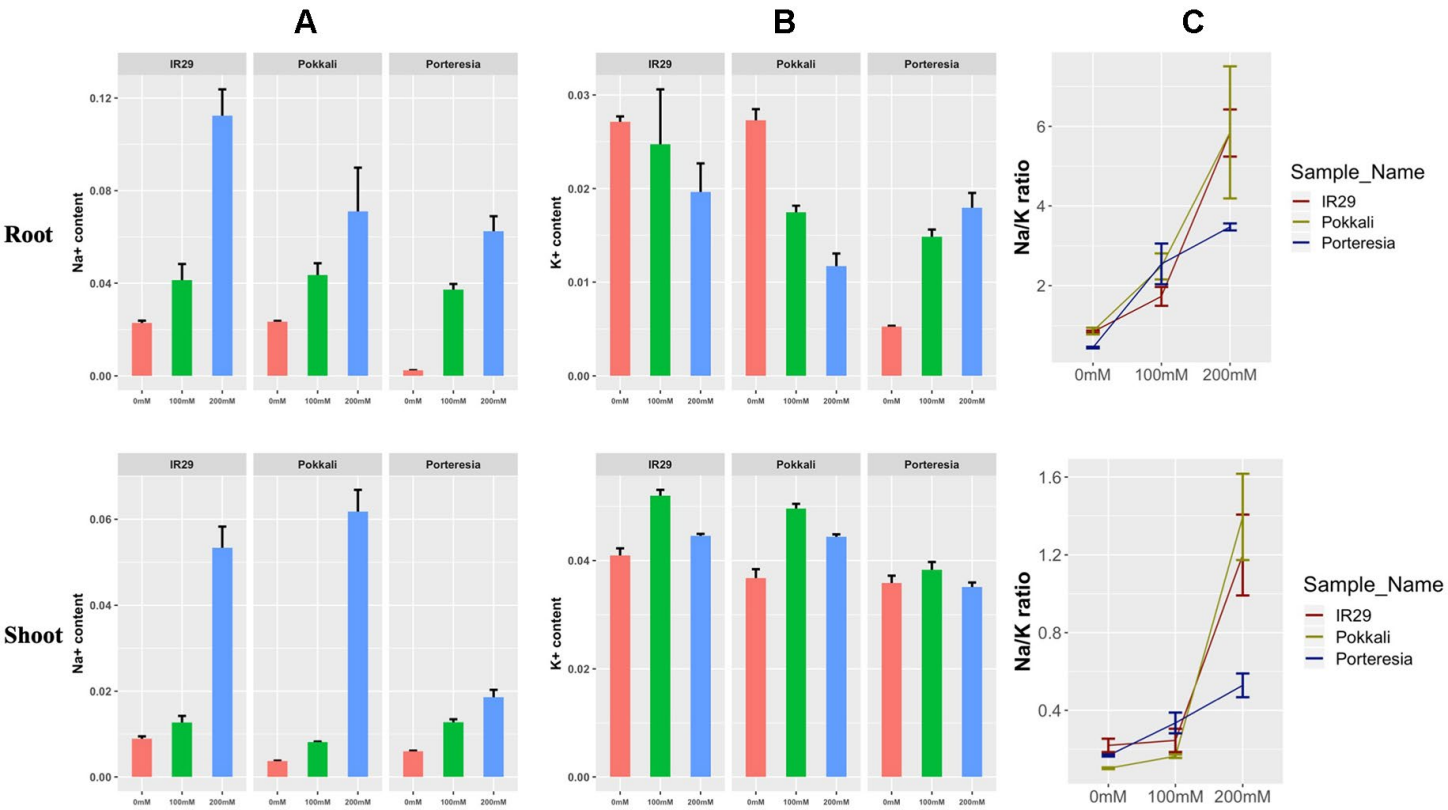
Different measurement of **(A)** Na^+^ content, **(B)** K^+^ content, and **(C)** Na^+^/K^+^ ratio under 100 and 200 mM salt stress conditions at 24 h in IR29, Pokkali, and *P. coarctata* rice variety in root and shoot. The measurements were taken from three biological replicates for each condition.

At 24 h, the Na^+^ content was found to increase in salt sensitive IR29 under 100 and 200 mM salt stress in root and shoot, but the pattern was slightly different to that of salt tolerant Pokkali (**Figure 4A**). Similarly K^+^ concentration was found to increase in shoots and decrease in roots both in IR29 and Pokkali, indicating transport of some K^+^ from root to shoot (**Figure 4B**). The red, green, and blue color denotes 0, 100, and 200 mM salt stress conditions in the figure. The ratio of Na^+^/K^+^ ratio was found to be lower for IR29 (denoted by dark red) compared to that of Pokkali (denoted by yellowish green) but it appeared that both plants were unable to maintain this lower ratio at the higher salt concentration (**Figure 4C**). On the contrary, *P. coarctata* (denoted by dark blue), can maintain lower Na^+^/K^+^ ratio in both roots and shoots under the higher stress of 200 mM after 24 h in contrast with that of IR29 and Pokkali (**Figure 4C**).

### Categorization of Retrieved HKT1;5 Sequences Based on Salt Tolerance

In this study, 23 sequences of *Oryza* genotypes among which 14 were retrieved as nucleotide sequences and 9 were obtained as amino acid sequences from NCBI databases. Among them 13 are considered as tolerant, 1 moderately tolerant, and 5 as sensitive varieties according to the literature cited below. Tolerance ability of *O. brachyantha* is unavailable (Walia et al., 2007; Platten et al., 2013; Mishra et al., 2016; Tiwari et al., 2016). PUSA basmati 44 has a score of 5 at the 10^th^ day of screening under 150 mM salt stress which was therefore considered as a moderately tolerant variety (Mishra et al., 2016). VSR 156 has a low susceptibility index value of 1.15 which indicates its high tolerance capability. For example, Pokkali, a tolerant variety has a susceptibility value of 4.35 (Mishra et al., 2016). New plant type varieties NPT rice was launched at IRRI to increase the yield potential through improvement of plant type which include several agronomic traits such as large panicles, few unproductive tillers from tropical japonica variety and for this reason these varieties can be categorized as sensitive to salt stress (Khush, 1995; Peng et al., 1999). The tolerance ability of the rest of the species is cited in **Table 1**.

### Multiple Sequence Alignment of Retrieved Proteins Reveal Substitution in Particular Sites of HKT1;5

Among the total of 23 sequences of HKT1;5 downloaded, 9 nucleotide sequences were translated to proteins using ORF Finder tool for further alignment and other analysis. ClustalW program in MEGA software generated many conserved regions between the sequences as well as various substitutions in different places. Among the many substitutions present, four (140 P/A/T/I, 184 H/R, D332H, V395L) were selectively present in all salt tolerant varieties as shown for Nona Bokra previously (Ren et al., 2005). Although Ren et al. showed substitution in 332 and 395 position but in our study due to different alignment parameter and gap opening the position changed to 333 and 396 respectively. But we will denote 332 and 395 in here for better understanding.

**Figure 5:**
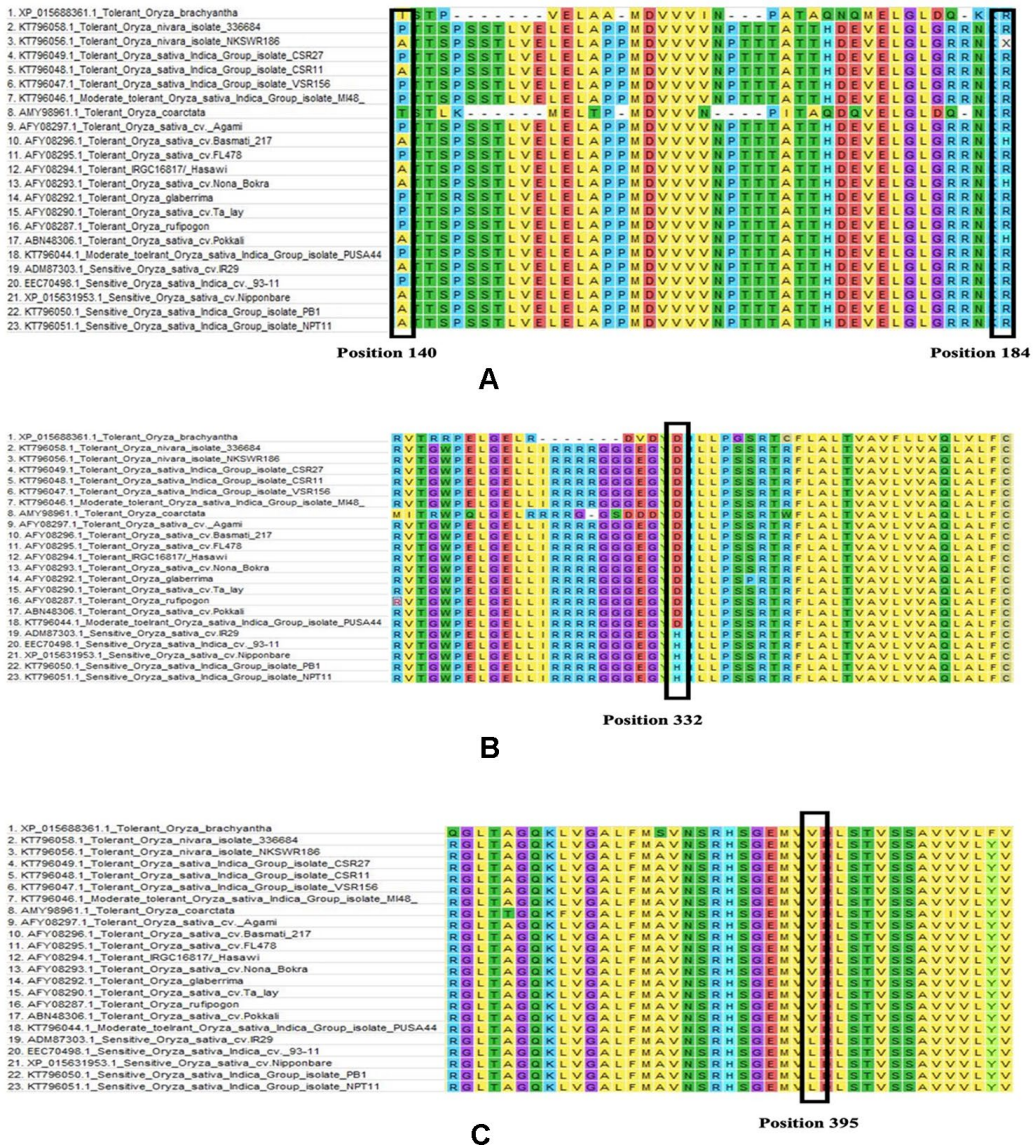
Multiple sequence alignment partial result of salt sensitive and salt tolerant rice varieties (**Table 1**). Amino acid sequence alignment results for **(A)** 140 to 184, **(B)** 310 to 360, and **(C)** 370 to 410 is provided. Changes in the position 140, 184, 332, and 39 has been marked with a black box. **(A)** In 140 position substitution is seen between aspartic acid, proline, isoleucine, and threonine and in 184 position arginine to histidine. **(B)** In 332 position aspartic acid to histidine substitution and **(C)** in 395 position valine to leucine substitution was observed. The latter two substitutions are present only in the sensitive genotypes.

From this alignment result, it can be observed that the first two substitutions (140 and 184) are not conserved. Alanine and Proline both are seen in salt tolerant and salt sensitive plants in position 140 whereas threonine and isoleucine is seen in *O. coarctata* and *Oryza brachyantha* respectively. Similarly, arginine is also present at position 184 in salt tolerant and salt sensitive plants alike. In some tolerant varieties, histidine is also seen in position 184 (**Figure 5**). Therefore, these substitutions appear to be inconclusive to serve any role in HKT1;5 for conferring salt tolerance in plants.

From our multiple alignment results, we found that aspartate substitution at 332 is conserved in addition to the valine at position 395 in all salt tolerant accessions, including the halophyte, wild salt tolerant rice, *O. coarctata* (**Figure 5**). Furthermore, we collected 137 accessions from the 3,000 rice genome project and checked for their SES score after 120 mM salt stress for 14 days as well as nucleotide substitution from RICE SNP-Seek database (Li et al., 2014; Mansueto et al., 2016). This shows that sensitive varieties have cytosine in 994 and 1,183 position (corresponding to His and Leu, respectively) whereas tolerant varieties have guanine (**Table 2** and **Supplementary Table 5**). Moreover, the presence of aspartate at 332 and valine at 395 position is observed in tolerant wheat TmHKT1;5 and TaHKT1;5 but due to their wide sequence diversity the amino acid positions were not the same and therefore they could be aligned over a short distance only (**Supplementary Figure 2**) (Munns et al., 2012).

**Table 2:** Nucleotide substitution between salt sensitive and tolerant variety.

*Variety*	*Position*			
	332 (994–996)		395 (1,183–1,185)	
*Tolerant*	Amino acid	Nucleotide	Amino acid	Nucleotide
*Sensitive*	Histidine	CAC	Leucine	CTC
*Tolerant*	Aspartic acid	GAC	Valine	GTC

### Phylogenetic Analysis Shows Close Evolutionary Relationship

**Figure 6:**
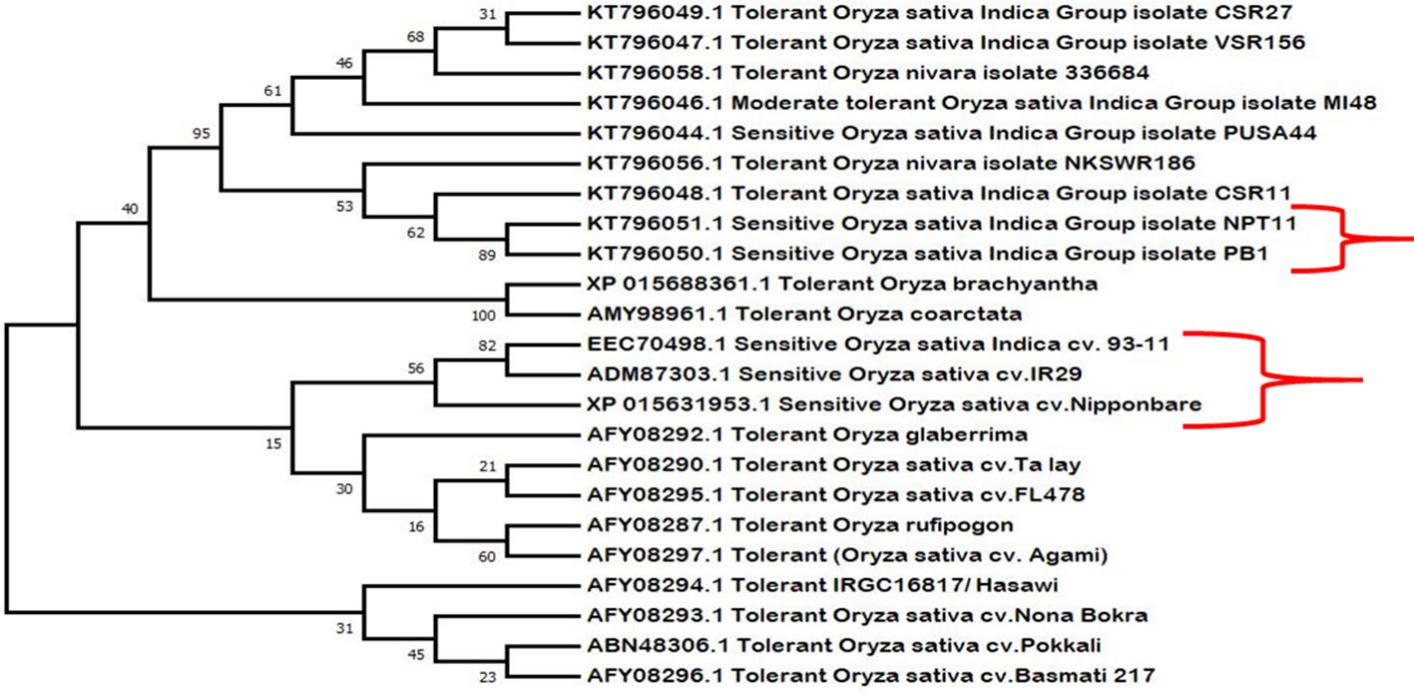
Phylogenetic analysis of the retrieved HKT1;5 protein sequences of salt sensitive and salt tolerant variety. Here salt sensitive and tolerant varieties remain clustered in separate clades, indicative of their close relatedness with respect to function. Species such as *Oryza coarctata* and *Oryza bhachyantha* also clustered together, which are distantly related otherwise. The sensitive varieties are marked using brackets “}”.

Phylogenetic analysis shows close evolutionary relationship among different HKT1;5 proteins (**Figure 6**). The salt sensitive plants and salt tolerant plants were grouped into separate clades showing that there are differences in tolerant and sensitive varieties. It was also observed that plants with distant relationships but which are salt tolerant (such as the halophyte *O. coarctata* and *Oryza glaberrima*) have the same substitutions that are found in tolerant rice. We have therefore proposed a hypothetic model to explain the role of two specific amino acids of HKT1;5 to maintain high Na^+^/K^+^ ratios in plants which show salt tolerance.

### Molecular Modeling and Structure Validation of HKT1;5

Quality of all the models (**Supplementary Figure 3**) are assessed using various methods and the results are summarized in **Supplementary Table 6**. The validation process of ERRAT is by statistical relation of non-bonded interactions among different atom types based on their characteristics atomic interactions with other atoms (Colovos and Yeates, 1993). This software assesses overall quality of the model with 0.01 and 0.05 level of significance. Generally high resolution structure models show overall quality to be 95% or higher and low resolution quality to be around 91% (Colovos and Yeates, 1993). Our predicted models show values from 67.78 to 84.94%. The best model was model 3 in this regard compared to others. Verify3D determines the compatibility of predicted model with own amino acid sequences by assigning a structural class based on its location and environment and results comparing it with good structure (Bowie et al., 1991). From this analysis, the best model was found to be model 3 with score of 70.06 compared to others. Rampage result of model 3 was found to be better than model 4.

Based on the sequence identity, GMQE, Verify3D, ERRAT Rampage results, the best model was chosen to be model 3 (template ID 4j7c.1.I) (**Supplementary Figure 4A**) compared to other plausible models (see *Materials and Method*).

The root mean square deviation (RMSD), which is the mean standard deviation of atomic coordinates between model and template structure, was found to be 2.768 Å using PyMol software where RMSD < 3 Å is taken as a good fit (Chothia and Lesk, 1986). In addition to this, the distribution of different amino acids in Ramachandran plot was checked to validate the structure (**Supplementary Figure 4B** and **Supplementary Table 7**). There are ~ 80% residues in the favored region which is not indicative of a good quality structure, yet one thing worth mentioning that the Ramachandran outliers are mostly in the large exterior loop region (~110–200). The sequence of residue 110–200 was also predicted to be extracellular loop by PSIPRED (using MEMSAT-SVM transmembrane helix prediction map) (Nugent and Jones, 2009; Buchan and Jones, 2019). Using such low sequence identity, proposition of a good model was never expected, rather the objective of the homology modeling was to get an overview of the structural arrangement of the protein inside the membrane, position of suggested critical residues (395Val and 332Asp) in the structure and be able to study dynamics to look at the regulation of those residues over ion permeation. The CELLO and PSIPRED results showed that the protein is a plasma membrane protein with eight transmembrane region validating earlier experiments that it stays in between xylem vessel and xylem parenchyma. The z-score was found to be -5.32.

### Aspartic Acid and Valine Positioned in the Opposite End of the Predicted 3D Structure of HKT1;5

**Figure 7:**
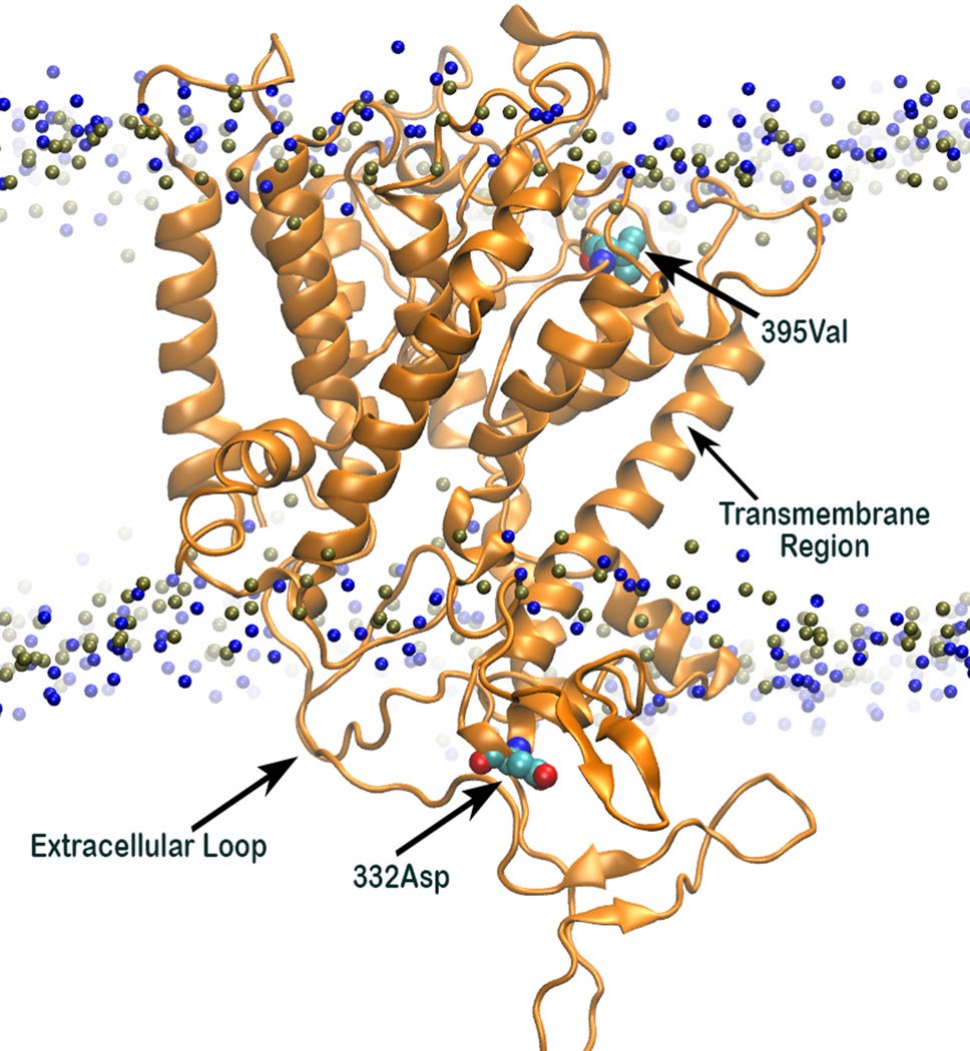
3D structure of Nona Bokra (orange cartoon) embedded in a membrane bilayer (membrane head-group P and N atoms are shown in blue and tan beads). The position of Val395 and Asp332 (shown in sticks) are just at the opposite end of the channel.

Analysis of 3D structure of HKT1;5 showed that Aspartic acid (at position 332) and valine (at 395 position) are in the opposite end of the embedded protein in the transmembrane region and close to the channel or pore for movement of either Na^+^ or K^+^, due to which substitution in this position has an immense effect on the functioning of the HKT1;5 protein (**Figure 7**). The position of valine HKT 1;5 is at the opening of the channel toward xylem vessel and aspartic acid is located close to the opening of the channel toward xylem parenchyma. Conservation of these positions among several sequences and their topological map along the channel structure seems to be strategic toward maintaining Na^+^/K^+^ and conferring salt tolerance ability.

### Narrow and Rigid Selectivity Filter for Sensitive Variety

**Figure 8:**
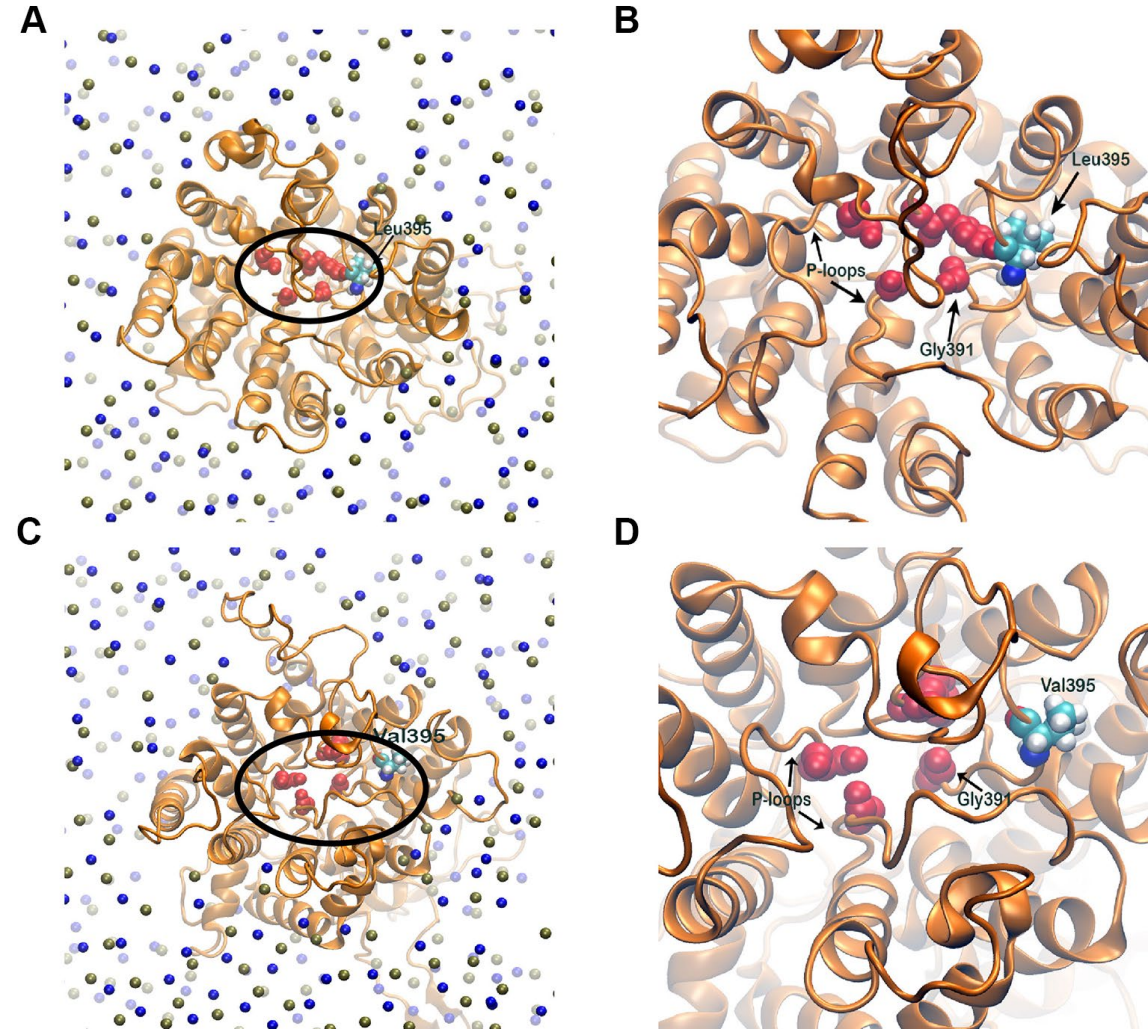
Membrane embedded structures (top view) of Nipponbare **(A)** and Nona Bokra **(C)**. The protein is shown in orange cartoon and membrane is represented using P (tan sphere) and N atoms (blue spheres). The selectivity filter residues are shown in red spheres and marked with black circle. These selectivity filter residues and residue 395 of both the proteins are amplified in **(B)** and **(D)**. The filter pore is narrower for Nipponbare compared to Nona Bokra.

**Figure 9:**
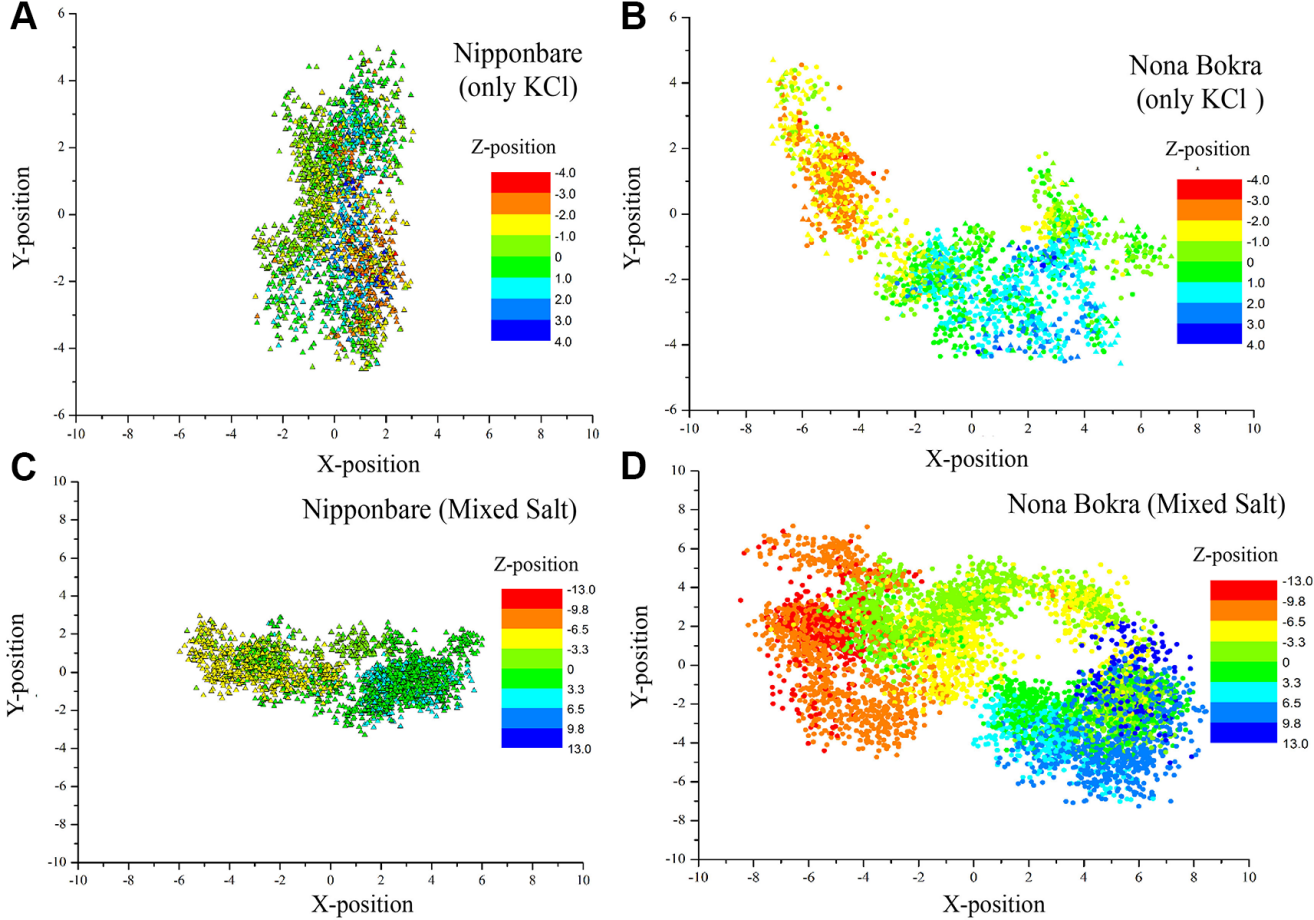
Centre of mass of side chain atoms of selectivity filter residues (Ser76, Gly264, Gly391 and Gly495) are calculated from last 20 ns of each trajectory and plotted in XY plane. Z-positions are projected on the XY plane using color gradient. All the X, Y, and Z distributions are centered around (X, Y, Z) = (0, 0, 0). **(A** and **B)** contain only KCl solution; **(C** , **D)** contain mixed salt solution (NaCl and KCl).

The selectivity filter of the HKT1;5 protein is made of Ser76, Gly264, Gly391, and Gly495 in both sensitive and tolerant variety (red spheres in **Figure 8**), which selectively transports the Na^+^ ion through the channel from xylem vessel to xylem parenchyma. As these four residues are mainly placed at four short loops called P-loops (Horie et al., 2001) present within the pore, dynamics of these loops can be crucial for the filter pore structure and dynamics. In addition, residue 395 is placed in the vicinity of one of the selectivity filter residue, Gly391 and as both share the same P-loop, dynamics of residue 395 itself can be critical to characterize the selectivity filter structure and dynamics. From molecular dynamics (MD) simulation trajectories, it was observed that the pore through selectivity filter is narrower (**Figures 8A, B**) in sensitive variety in comparison to that of the tolerant variety (**Figures 8C, D**). Not only that, visual inspection suggests that the selectivity filter residues were much flexible in tolerant variety in comparison to sensitive Nipponbare. This was further validated by plotting the centre of mass of side chain atoms, in selectivity filter residues from last 20 ns of each trajectory in XY plane and corresponding Z-positions using color gradient (**Figure 9**).

**Figure 10:**
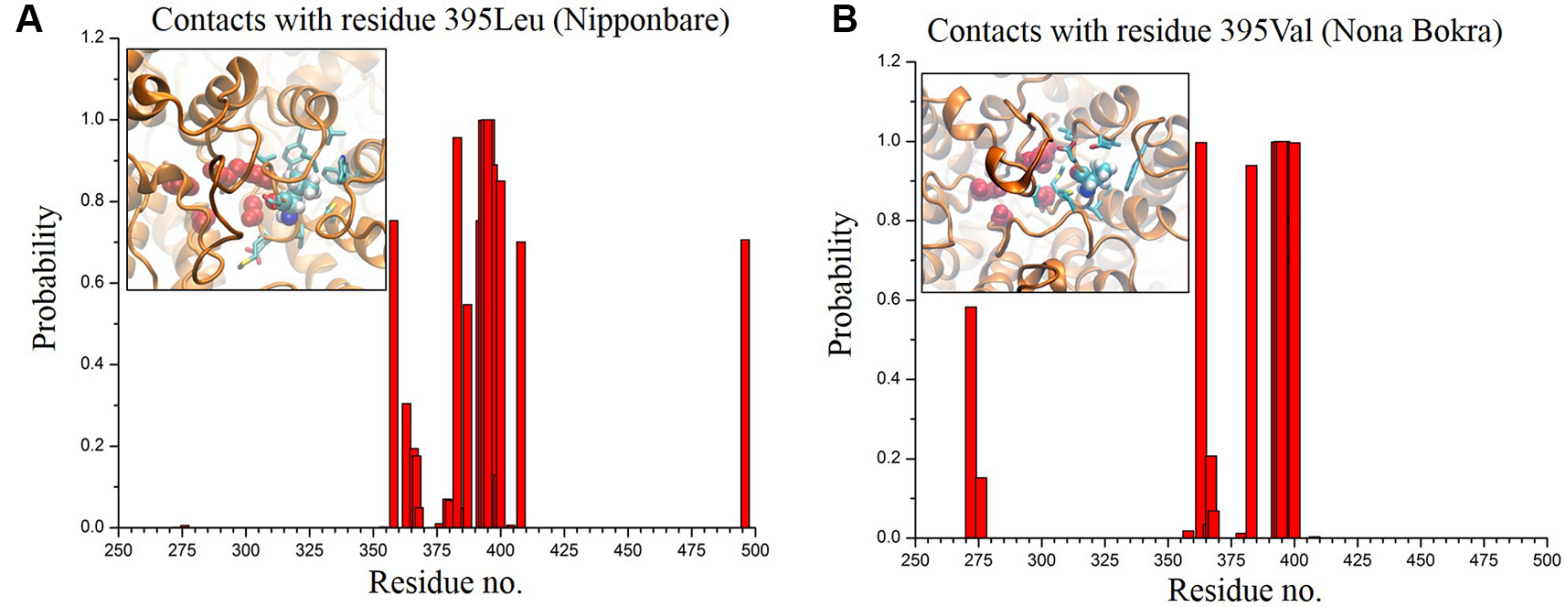
Contact probability of residue 395 with all the other residues of Nipponbare **(A)** and Nona Bokra **(B)**. There are more number of local contacts (residue ~ 350–410) with significant probability involving 395Leu in comparison to Nona Bokra. Local crowding around residue 395 is represented in inset of the probability plots following the same representation of **Figure 10**. Surrounding residues are shown in cyan sticks.

In only KCl solution, there isn’t much difference in Z-axis spread. In XY-plane, Nipponbare (salt sensitive) fluctuates mostly in Y-axis (**Figure 9A**) whereas Nona Bokra (salt tolerant) residues are spread over a much broader region (**Figure 9B**), relatively. For the systems in mixed salt solution (**Figures 9C, D**), the distributions brings much more clarity to the difference in selectivity filter dynamics, where Nipponbare (salt sensitive) residue distribution is rigid and spatially dense (**Figure 9C**), in comparison to the much broader fluctuation of Nona Bokra (salt tolerant) residues in all the directions (**Figure 9D**). These results are all indicative of a more rigid and narrow pore of Nipponbare (salt sensitive) in comparison to Nona Bokra (salt tolerant). This flexible and wide passage through the selectivity filter of Nona Bokra (salt tolerant) is expected to cause Na^+^ efflux more easily in comparison to the rigid and narrow pore of Nipponbare (salt sensitive). However, it is not clear yet that why such difference appears between these two varieties. As mentioned earlier, this could be due to the residue at position 395. The contact probability of residue 395 with all the other residues has been calculated over the trajectory of KCl solution systems and plotted in (**Figure 10**). Formation of contact is defined if any two heavy atoms from any two residues are within 4.2 Å of each other and the probability is calculated as the fraction of time a contact was stable in a trajectory, and so it varies from 0 (that contact was not formed even once between two residues) to 1 (contact was stable throughout the trajectory). In Nipponbare, 395Leu is able to make contact with many hydrophobic residues (e.g. 358Phe, 363Trp, 383Met, 408Tyr, 496Phe) with significant stability (**Figure 10A**). This network may impose a restriction to the P-loop which accommodates 395Leu as well as one of the selectivity filter residue, i.e. 391Gly. So, restriction in the P-loop may add rigidity to the flexibility of the selectivity filter. In contrast, though there are few stable contacts (e.g. 272Leu, 363Trp, 383Met), 395Val of Nona Bokra is unable to make efficient stable hydrophobic contacts due to its reduced side chain length (**Figure 12B**). That can be the reason for flexible and wide pore through selectivity filter residues. The rigidity imposed by position 395 has also been suggested previously (Cotsaftis et al., 2012).

### Extracellular Loop Motion Regulated by Residue 332

**Figure 11:**
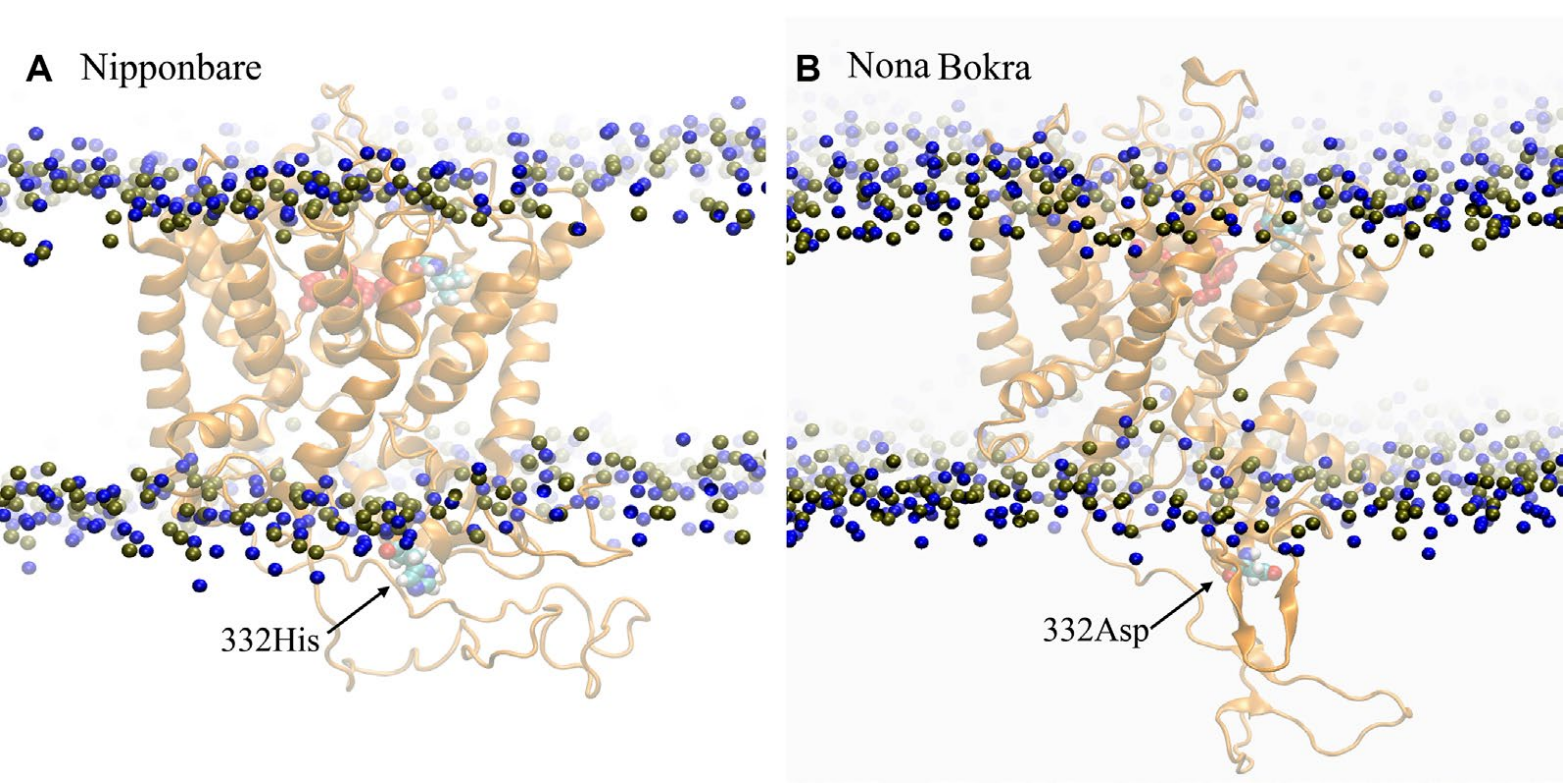
Representative conformation of the extracellular loop in **(A)** Nipponbare and **(B)** Nona Bokra. Representation is similar to previous graphics.

**Figure 12:**
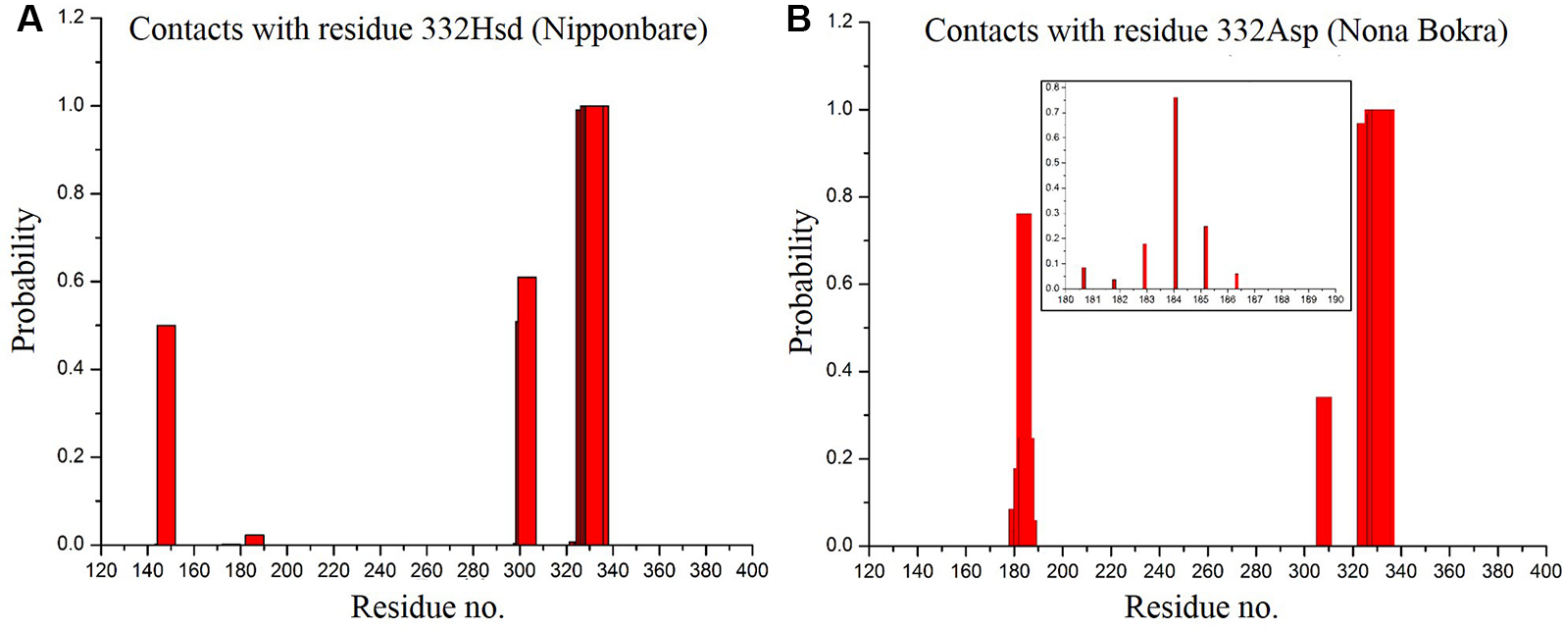
Contact probability of residue 395 with all the other residues of Nipponbare **(A)** and Nona Bokra **(B)**. Contact of 332 Asp with loop residues are shown in inset of **(B)**.

According to our model, residue 332 lies around the constriction pore, and in close proximity to the large extracellular loop (residue ~110–200). Considering the fact that the confidence on the model is not satisfactory, the trajectory showed that the dynamics of the large loop around the constriction pore can be important for the passage of ions. As the loop consists of many polar residues, it can trap ions if it folds around the pore opening as it appeared to be the case for Nipponbare (**Figure 11A**). This can make the passage of Na^+^ comparatively difficult for Nipponbare which can be severe under salt stress. On the other hand, in case of Nona Bokra, the loop is more dynamic and away from the pore opening (**Figure 11B**) which can be beneficial to the efflux of Na^+^ under any condition. Again, contact probability was calculated between residue 332 and other residues and plotted in **Figure 12**. In Nipponbare, 332His interacts locally with many surrounding residues and is not involved in many distant interactions (**Figure 12A**) due to its smaller size and rigidity. On the contrary, 332Asp of Nona Bokra is flexible and polar and thus involved in significant interactions with many polar residues (~ residue 180–190) of the loop. It cannot be concluded here that the involved polar contacts are exclusively involved in the swap out of the loop from the vicinity of pore opening, yet this can be one possible reason for the distinct loop dynamics.

## Discussion

### HKT1;5 Mediate Na^+^ Unloading From Xylem Vessels in Roots Dominantly Under Salt Stress

HKT1;5 is a vital transporter protein that recirculates Na^+^ to maintain the homeostasis of Na^+^/K^+^ in higher plants (Schachtman and Schroeder, 1994; Munns and Tester, 2008; Horie et al., 2009). In rice this transporter is identified as the mediator for Na^+^ retrieval from xylem to xylem parenchyma in rice (Ren et al., 2005) and wheat (Byrt et al., 2007). Our study based on microarray data, mRNAseq data and real time expression analysis showed that its presence is more in root compared to that of the shoot. One group showed that, OsHKT1;5 helps in the exclusion of Na^+^ from leaves by transferring Na^+^ from xylem sap in roots (Cotsaftis et al., 2012). In wheat TmHKT1;5-A and TaHKT1;5-D found to be expressed in roots but not in leaves and their gene expression gradually increased with higher levels of salt stress (Byrt et al., 2007; Munns et al., 2012). Our current study results with IR29 (salt sensitive), Pokkali (salt tolerant), and *Porteresia coarctata* (halophyte) showed that HKT1;5 is expressed mainly in roots and it was upregulated by 100 mM salt stress. The increase in expression of the *P. coarctata* transporter was comparatively much higher with the gradual increase (100 and 200 mM) of salt stress indicating its important role during stress tolerance in the recirculation process of Na^+^. Although Pokkali and *P. coarctata* are able to survive in 100 mM stress, IR29 cannot, despite showing similar increase at 24 h in relative expression change of 2.4 fold comparable to Pokkali.

### Balancing Na^+^ and K^+^ Ions Under Salt Stress Is Crucial for Plant Survival

The survival of plants largely depends on the balancing of Na^+^/K^+^ although the complete mechanism involved in this process are still not clearly elucidated. With the increase of salt stress, the salt sensitive and tolerant variety try to mediate the homeostasis of Na^+^ by retrieving it from xylem vessel into xylem parenchyma by HKT1;5 (Ren et al., 2005). It was observed here that despite similar gene expression change, sensitive IR29 is unable to survive, at 100 mM stress, while tolerant Pokkali does. Higher Na^+^ content was found in the shoot in IR29 compared to Pokkali. This indicates that Na^+^ could not recirculate from shoot to root easily in IR29 as opposed to the situation in Pokkali leading to higher Na^+^/K^+^ ratio in the shoot of the former. This points to species-specific Na^+^ toxicity threshold that would hamper normal functioning in shoot (Maggio et al., 2001; Chen et al., 2007). Plants that are continuously exposed to their habitat-specific coastal salt such as the halophyte *P. coarctata*, however has the ability to maintain low cytosolic Na^+^/K^+^ ratio in presence of high salt stress which is explained by the continued overexpression of HKT1;5 with the gradual increase in salt stress. Previous studies showed that, under 120 mM salt stress for 7 days, salt tolerant varieties were found to be able to maintain better Na^+^/K^+^ ratio in shoots compared to salt sensitive ones (**Supplementary Figure 1**, data collected from DeLeon et al. (2015). These results are also corroborated in earlier studies which demonstrated that growth and yield of plants are correlated with toxic ion accumulation in shoots but not in roots (Dalton et al., 2000).

An important question therefore arises whether the change in gene expression is the only vital factor for the survival of these plants or whether specific amino acids are playing important roles within the transporter protein structure.

### Two Amino Acid Substitution Model of the Oryza Species HKT1;5 Transporter

**Figure 13:**
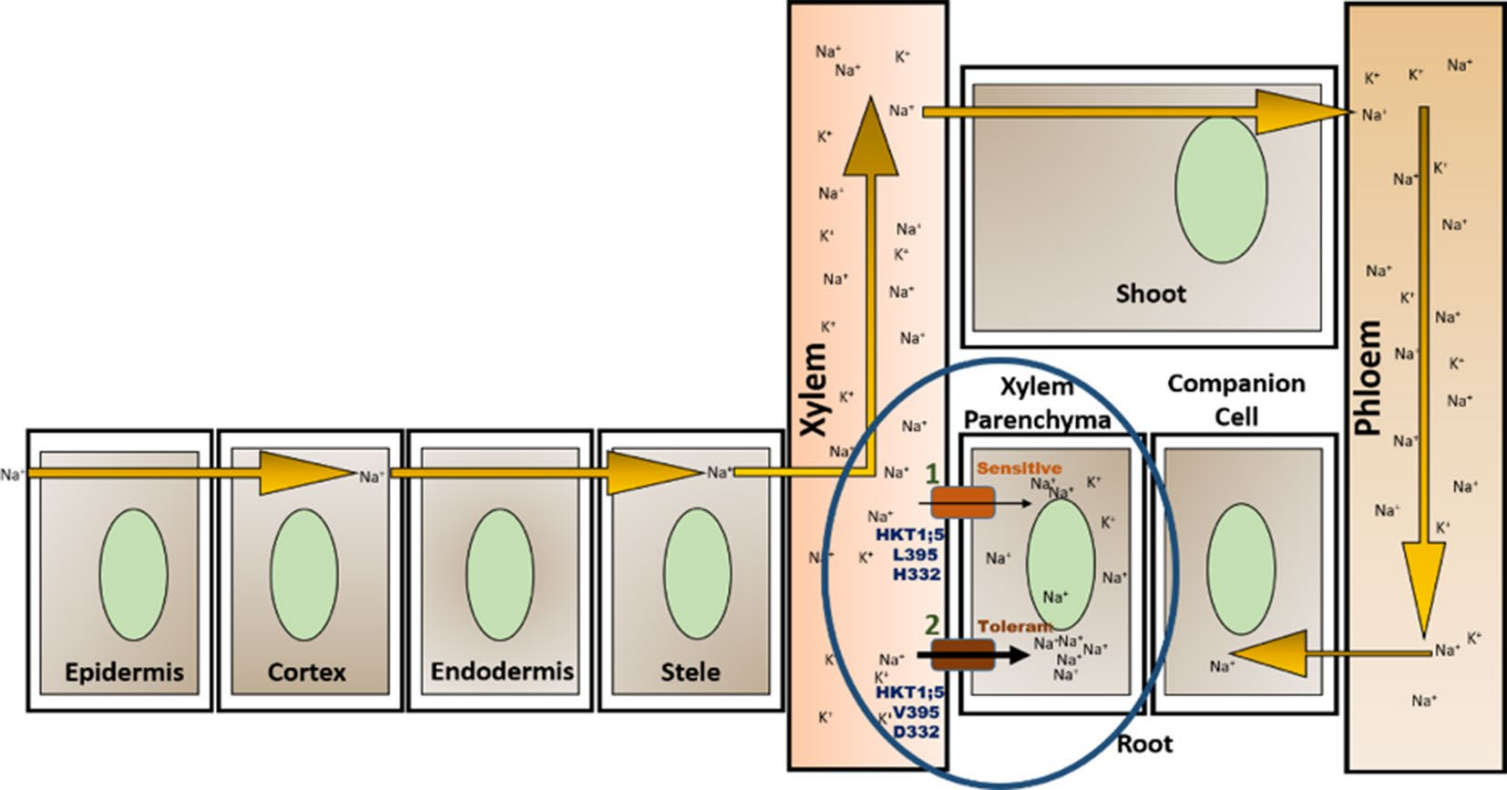
Location of OsHKT1;5 (circled) and two amino acid substitutions working together to give tolerance against salt stress. High salt concentration in soil allows Na^+^ to enter through root and transport throughout the plant *via* the xylem vessel and is recirculated back to the root through phloem (Maathuis, 2013). OsHKT1;5 functions by transporting Na^+^ out of xylem vessel into xylem parenchyma (efflux) minimizing the harmful effects to the plant due to Na^+^ accumulation. Presence of valine instead of leucine in position 395 and of aspartate in place of histidine (at position 332) allows for greater transfer rate of Na^+^ out of xylem vessel into the root xylem parenchyma (1, 2).

The 3D structure shows that the position of the two substitutions (395Leu/Val and 332Asp/His) are in the opposite end of the transporter channel (**Figure 7**). Strategic position of these two amino acids might be playing a decisive role for the tolerant variety to survive but not in sensitive varieties. The selectivity filter of the HKT1;5 protein consist of 76Ser-264Gly-391Gly-496Gly in both Nona Bokra (salt tolerant) and Nipponbare (salt sensitive), and all are accommodated in four P-loops (Horie et al., 2001). This filter selectively transports Na^+^ across the membrane. Results from the MD simulation study show that Val/Leu at the 395 position is crucial to the dynamics of selectivity filter as 391Gly (filter residue) shares the same loop as 395Val/Leu. In sensitive variety, 395Leu forms an intricate residual network where many of the residual contacts are stabilized by Van der Waals interaction with the surrounding hydrophobic amino acids. This network possibly restricts the loop motion which would eventually reduce flexibility of 391Gly. This could be the possible reason for the narrow and rigid pore in sensitive variety, i.e. Nipponbare. Whereas, in tolerant variety (Nona Bokra), presence of Valine makes the positon 395 less prone to get stabilized by hydrophobicity which causes more flexibility to the subsequent P-loop and eventually to Gly391. This probably makes the selectivity filter more flexible and wide for favorable permeation of Na^+^ as suggested by Cotsaftis (Cotsaftis et al., 2012). In rice, this would translate into more flexible movement of Na^+^ from xylem sap into xylem parenchyma in tolerant variety (**Figure 13**) leading to its net decrease in the transpiration stream and resulting in lower toxicity in plants. This Na^+^ eventually moves out of root cell into the soil in all likelihood, by the help of other transporters such as SOS1.

This standalone model however may not provide the full picture unless we discuss another important substitution (i.e. at position 332) that probably plays an important role as well, in the functioning of HKT1;5 under high salt stress.

The presence of 332 positon is at the opening of HKT1;5 to the xylem parenchyma part of plant root. Sequence alignment showed that this position has either histidine (sensitive) or aspartic acid (tolerant) and this is in the vicinity of large extra cellular loop, enriched with polar residues, at the parenchyma side of the root (**Figure 11**). In a sensitive variety, presence of histidine 395 leads to few local and less polar interactions with the surrounding amino acids causing the loop residues to spread out. The loop can freely fold and get lodged around the opening of constriction pore due to polar interaction with the membrane polar head-groups (**Figure 11A**). This would eventually create hindrance in the clearance of Na^+^ that has already transported through the pore from the opposite end. In contrast to that, aspartic acid in Nona Bokra (salt tolerant) at the same position, can get involved in polar interaction with the loop residues, impacting the loop dynamics in such a way that that it shifts away from the vicinity of the constriction pore and makes the efflux of Na^+^ easier. Transportation of Na^+^ into xylem parenchyma from the xylem sap causes membrane depolarization activating some transporters such as SKOR (stelar K^+^ outward rectifying), OsHAK5, OsHAK1, OsAKT, thus allowing K^+^ to be transported in the opposite direction and into the xylem sap (Very and Sentenac, 2003; Assaha et al., 2017). This movement of K^+^ would in effect maintain the Na^+^/K^+^ ratio.

The structural confidence of our model is not high due to lower level of similarity between the template and predicted protein. However, in absence of solved protein structures of this family of proteins and subsequent experimental data, the present approach can be important to get an overview of a plausible mechanism of action for the HKT1;5 transporter. Notwithstanding such limitations, the present study which suggests a model based on two amino acid substitutions working together to enable salt tolerant varieties to remove toxic Na^+^ and maintain a low Na^+^/K^+^ ratio seems possible. Firstly, the presence of 395Val at the side of xylem vessel enables Na^+^ efflux through a wide and flexible pore. Secondly, the presence of 332Asp at the side of xylem parenchyma of root clears the way for the already inserted Na^+^ from the xylem vessel end by controlling the dynamics of extracellular loop. The Na^+^ can then move out of the cell through the SOS1 transporter. These two amino acid substitutions working together in sync may enable salt tolerant varieties to survive in the salt stress environment as modeled.

## Conclusion

Functional and structural studies regarding OsHKT1;5 transporter genes and proteins are limited. In this study we have analyzed gene expression of IR29, Pokkali, and *P. coarctata* to check the tissue specificity, relative expression ability of Na^+^/K^+^ ratio maintenance. Although the expression could be checked under longer time interval which might provide a detailed response pattern of HKT1;5. Further, we have analyzed 23 sequences collected from NCBI and 137 sequences from 3,000 genome project of salt tolerant, salt sensitive and their wild relative rice genotype. Our study revealed that the two substitutions, working synchronously, namely, leucine to valine in position 395 and histidine to aspartate in position 332 only in the tolerant genotypes including a distant halophyte may have an important function in providing salt tolerance. However, this will need to be validated experimentally. In phylogeny analysis a clear distinction of OsHKT1;5 between salt sensitive and salt tolerant varieties was also observed. As more sequences of HKT1;5 genes, including those of halophytes are collected and compared, the importance of these two amino acids in distinguishing between salt sensitive and tolerant genotypes can be further confirmed and validated in future studies. It may be pointed out here that while comparing distant species the localized positioning of two amino acids within the membrane may be more important than overall sequence alignment. Thus, the introduction of aspartate replacing histidine and valine replacing leucine in HKT1;5 transporters proposes a model for an altered ion selectivity and uptake kinetics. This change in two amino acids may help species with a particular lineage acquire improved salt tolerance over a long evolutionary period.

## Data Availability Statement

All datasets generated for this study are included in the article/**Supplementary Material**.

## Author Contributions

Project supervision: ZS. Idea generation: MS, ZS. Data curation: MS. Formal analysis: MS, SS. Methodology: MS, SS. Na+/K+ content estimation: FN. Simulation study: SS, MS. Supervision of simulation study: SD. Writing–original draft: MS, ZS, SS, SD.

## Conflict of Interest

The authors declare that the research was conducted in the absence of any commercial or financial relationships that could be construed as a potential conflict of interest.

## Supplementary Material

Supplementary Figure 1 Na+/K+ ratio of salt senstitive( green) and salt tolerant (blue) varieties under 120mM salt stress for 7 days.

Supplementary Figure 2 Multiple Sequence alignment partial result of 3 salt sensitive, 3 salt tolerant rice and 2 tolerant wheat varieties. Amino acid sequence alignment results for 324 to 404 is provided. The presence of Aspartate at 332 and Valine at 395 position is also observed in tolerant wheat TmHKT1;5 and TaHKT1;5.

Supplementary Figure 3 Different models of HKT1;5 generated based on different templates.

Supplementary Figure 4 Structure validation (a) 3D structure best predicted model of HKT1;5 (b) Ramachandran plot of the model structure (c) Using ProSA protein structural analysis tool the Z-score was found to be -5.32.
