# Supplementary figures and images for: HKT1;5 Transporter Gene Expression and Association of Amino Acid Substitutions With Salt Tolerance Across Rice Genotypes
Source: Front Plant Sci. 2019 Nov 4;10:1420. doi: 10.3389/fpls.2019.01420 (PMC6843544; doi:10.3389/fpls.2019.01420)

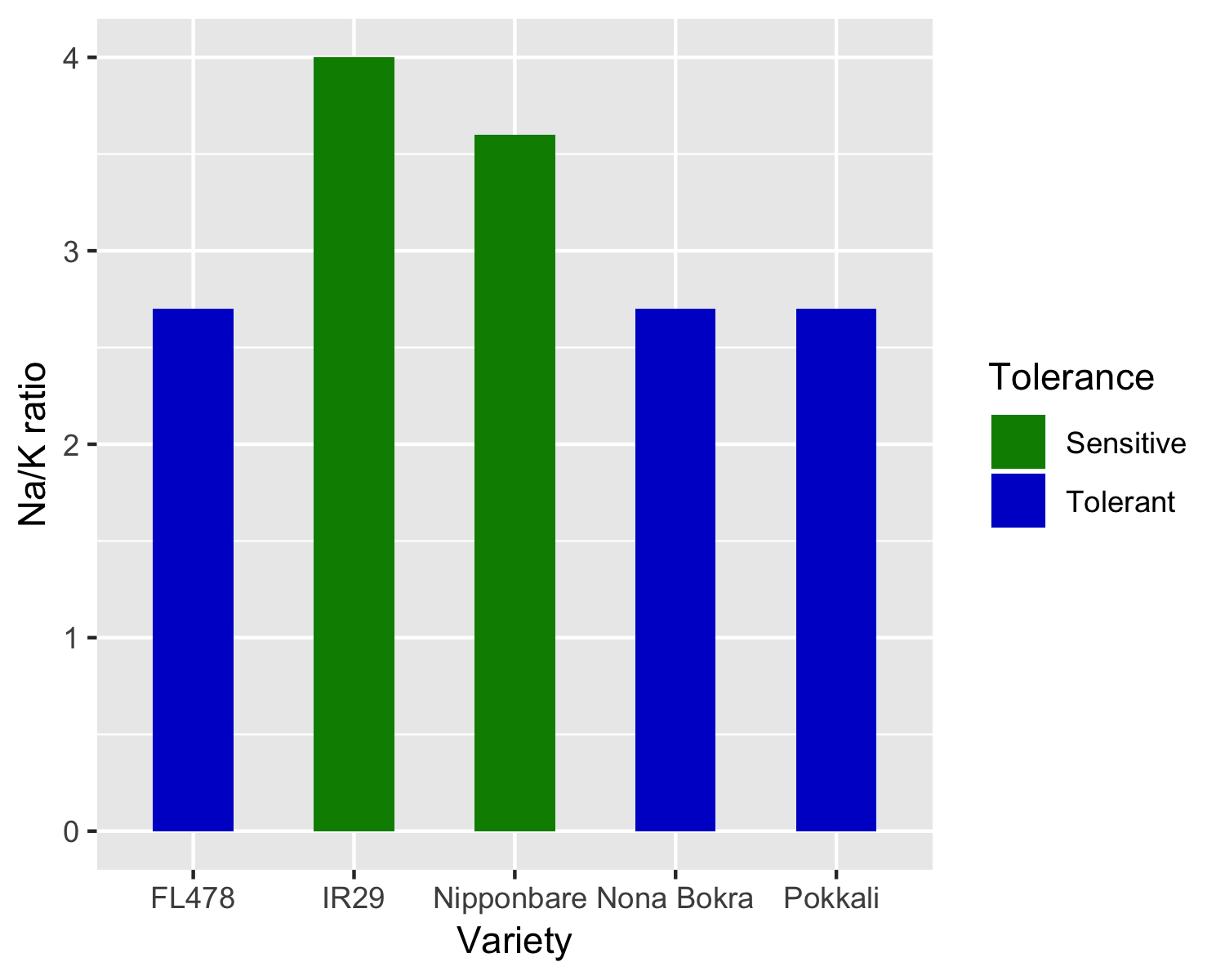

Supplement: Supplementary Figure 1 — Na+/K+ ratio of salt senstitive( green) and salt tolerant (blue) varieties under 120mM salt stress for 7 days. [file Image_1.tiff]

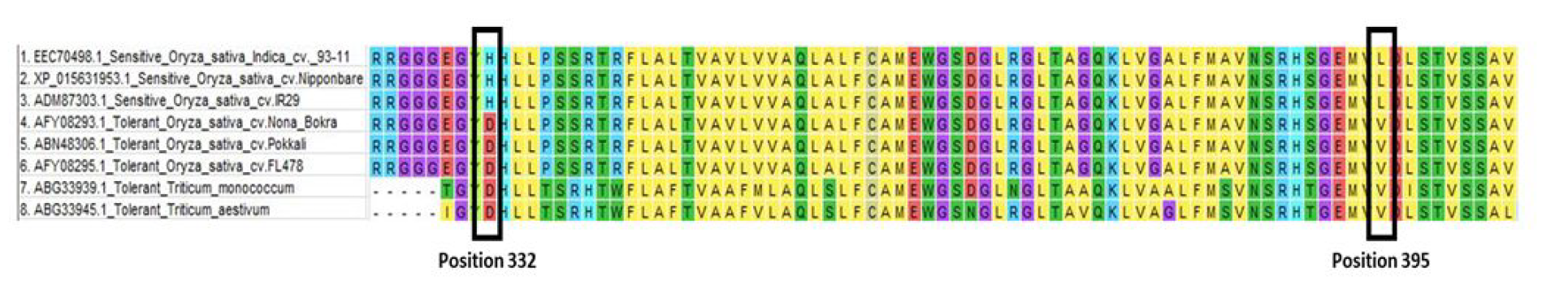

Supplement: Supplementary Figure 2 — Multiple Sequence alignment partial result of 3 salt sensitive, 3 salt tolerant rice and 2 tolerant wheat varieties. Amino acid sequence alignment results for 324 to 404 is provided. The presence of Aspartate at 332 and Valine at 395 position is also observed in tolerant wheat TmHKT1;5 and TaHKT1;5. [file Image_2.tif]

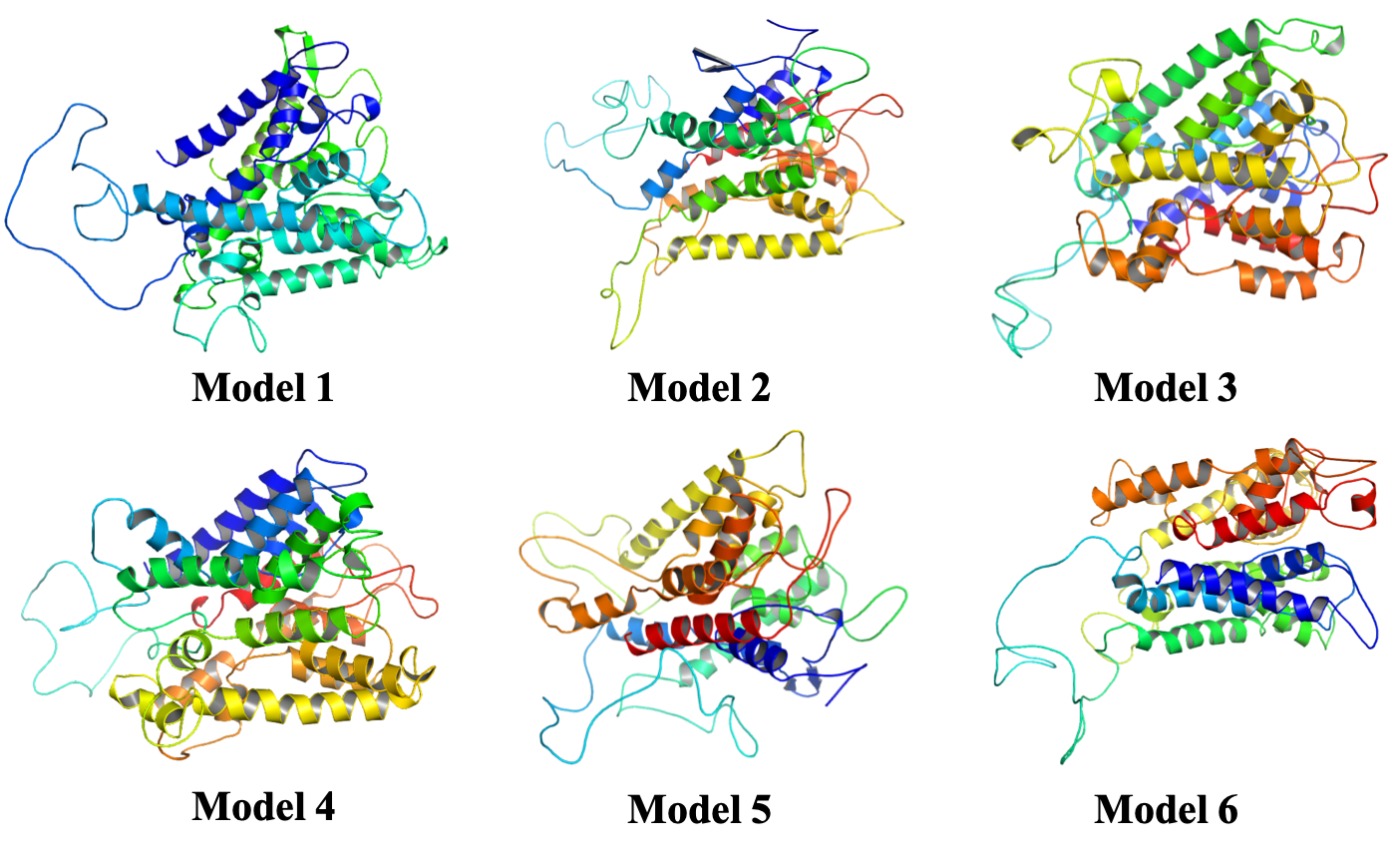

Supplement: Supplementary Figure 3 — Different models of HKT1;5 generated based on different templates. [file Image_3.tif]

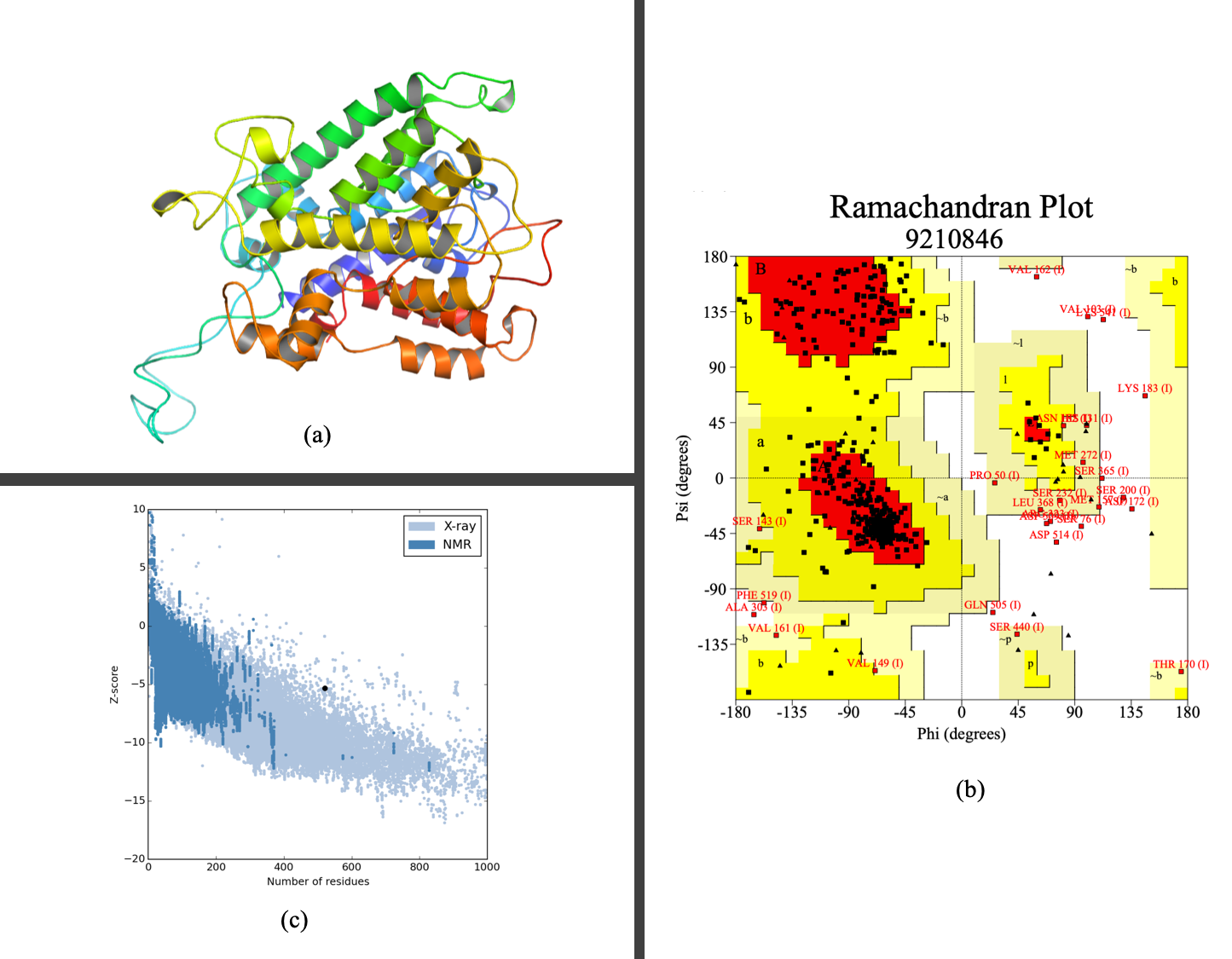

Supplement: Supplementary Figure 4 — Structure validation (a) 3D structure best predicted model of HKT1;5 (b) Ramachandran plot of the model structure (c) Using ProSA protein structural analysis tool the Z-score was found to be -5.32. [file Image_4.tif]
