# Supplementary material for: HKT1;5 Transporter Gene Expression and Association of Amino Acid Substitutions With Salt Tolerance Across Rice Genotypes
Source: Front Plant Sci. 2019 Nov 4;10:1420. doi: 10.3389/fpls.2019.01420 (PMC6843544; doi:10.3389/fpls.2019.01420)
Supplement: Supplementary file 5 [file Table_1.docx]

#### **Supplementary Table 1**: List of gene specific primers used in quantitative Real-Time gene expression analysis.

| Primer Name | Sequence | Length | Tm |
| --- | --- | --- | --- |
| EF_1α_F | TTTCACTCTTGGTGTGAAGCAGAT | 24 | 65.1 ̊C |
| EF_1α_R | GACTTCCTTCACGATTTCATCGTAA | 25 | 63.4 ̊C |
| OsHKT1;5_F | CCTGCCACCTTACACCACTT | 20 | 64.6 ̊C |
| OsHKT1;5_R | GCTGTAGTTGATGGGGTCGT | 20 | 64.2 ̊C |
| PcHKT1;5_F | CAGGTTTCAGAGGACCTACCACCTT | 25 | 67.3 ̊C |
| PcHKT1;5_R | GACGTGAAGATTAGGTCCAAGTCCA | 25 | 65.9 ̊C |
