# Supplementary material for: HKT1;5 Transporter Gene Expression and Association of Amino Acid Substitutions With Salt Tolerance Across Rice Genotypes
Source: Front Plant Sci. 2019 Nov 4;10:1420. doi: 10.3389/fpls.2019.01420 (PMC6843544; doi:10.3389/fpls.2019.01420)
Supplement: Supplementary file 6 [file Table_2.docx]

**Supplementary Table 2:** GEO accession of the mRNAseq data used in Genevestigator software

| Biotic | Chemical | Other | Photoperiod | Stress |
| --- | --- | --- | --- | --- |
| GSE81906 | GSE89494 | GSE73609 | GSE92302 | GSE78972 |
| GSE62488 |  | GSE89233 | GSE78972 | GSE92989 |
| GSE67958 |  | GSE95200 |  | GSE57950 |
| GSE67588 |  | GSE77300 |  |  |
|  |  | GSE73609 |  |  |
