# Supplementary material for: HKT1;5 Transporter Gene Expression and Association of Amino Acid Substitutions With Salt Tolerance Across Rice Genotypes
Source: Front Plant Sci. 2019 Nov 4;10:1420. doi: 10.3389/fpls.2019.01420 (PMC6843544; doi:10.3389/fpls.2019.01420)
Supplement: Supplementary file 7 [file Table_3.docx]

**Supplementary Table 3**: GEO accession of the microarray data used in Genevestigator software.

| Biotic | Chemical | Elicitor | Hormone | Light quality | Nutrient | Other | Photoperiod | Stress | Temperature |
| --- | --- | --- | --- | --- | --- | --- | --- | --- | --- |
| GSE19024 | GSE25206 | GSE25206 | GSE37557 | GSE36320 | GSE48549 | GSE35984 | GSE28124 | GSE45724 | GSE19024 |
| GSE32426 | GSE4471 | GSE30583 | GSE5167 |  | GSE17245 | GSE31834 | GSE29820 | GSE6908 | GSE54243 |
| GSE30942 | GSE53564 |  | GSE19024 |  | GSE38102 | GSE43780 |  | GSE6901 |  |
| GSE21919 | GSE25206 |  | GSE6719 |  | GSE85765 | GSE10857 |  | GSE33204 |  |
| GSE7256 |  |  |  |  | GSE17245 | GSE46342 |  | GSE37940 |  |
| GSE28308 |  |  |  |  | GSE35984 | GSE53347 |  | GSE38023 |  |
| GSE30941 |  |  |  |  | GSE38871 |  |  | GSE6901 |  |
| GSE21772 |  |  |  |  | GSE37161 |  |  | GSE42683 |  |
| GSE18361 |  |  |  |  | GSE44250 |  |  | GSE41647 |  |
| GSE30941 |  |  |  |  |  |  |  | GSE57154 |  |
| GSE18361 |  |  |  |  |  |  |  | GSE24048 |  |
| GSE29967 |  |  |  |  |  |  |  | GSE26280 |  |
| GSE80246 |  |  |  |  |  |  |  | GSE25176 |  |
| GSE74106 |  |  |  |  |  |  |  | GSE23211 |  |
| GSE22538 |  |  |  |  |  |  |  | GSE81253 |  |
| GSE11025 |  |  |  |  |  |  |  | GSE83378 |  |
| GSE10373 |  |  |  |  |  |  |  | GSE14275 |  |
| GSE33204 |  |  |  |  |  |  |  | GSE33204 |  |
| GSE16793 |  |  |  |  |  |  |  | GSE3053 |  |
| GSE33411 |  |  |  |  |  |  |  | GSE13735 |  |
| GSE19844 |  |  |  |  |  |  |  | GSE16108 |  |
| GSE36272 |  |  |  |  |  |  |  | GSE14403 |  |
| GSE33204 |  |  |  |  |  |  |  | GSE58603 |  |
| GSE36093 |  |  |  |  |  |  |  | GSE18930 |  |
| GSE34192 |  |  |  |  |  |  |  | GSE74106 |  |
| GSE43050 |  |  |  |  |  |  |  |  |  |
| GSE69235 |  |  |  |  |  |  |  |  |  |
| GSE19239 |  |  |  |  |  |  |  |  |  |
