# Supplementary material for: HKT1;5 Transporter Gene Expression and Association of Amino Acid Substitutions With Salt Tolerance Across Rice Genotypes
Source: Front Plant Sci. 2019 Nov 4;10:1420. doi: 10.3389/fpls.2019.01420 (PMC6843544; doi:10.3389/fpls.2019.01420)
Supplement: Supplementary file 8 [file Table_4.docx]

**Supplementary Table 4:** Study sample and GEO accession of the microarray data used to check differential expression of microarray data

| Study | GEO accession |
| --- | --- |
| salt study1(FL478) | GSE3053 |
| salt study1(IR29) | GSE3053 |
| salt study2(3h) | GSE6901 |
| salt study3(FL478) | GSE13735 |
| salt study3(IR29) | GSE13735 |
| salt study4(salt sensitive RILs) | GSE16108 |
| salt study4(salt sensitive RILs) | GSE16108 |
| salt study5(IR63731) | GSE14403 |
| salt study5(Pokkali) | GSE14403 |
| salt study7(IR64; ABA; root) | GSE58603 |
| salt study7(IR64; ABA; root) | GSE58603 |
| salt study7(IR64; ABA; shoot) | GSE58603 |
| salt study7(IR64; ABA; shoot) | GSE58603 |
| salt study7(IR64;root) | GSE58603 |
| salt study7(IR64;shoot) | GSE58603 |
| salt study7(PL177; ABA; root) | GSE58603 |
| salt study7(PL177; ABA; root) | GSE58603 |
| salt study7(PL177; ABA; shoot) | GSE58603 |
| salt study7(PL177; ABA; shoot) | GSE58603 |
| salt study7(PL177; root) | GSE58603 |
| salt study7(PL177; shoot) | GSE58603 |
