# Supplementary material for: HKT1;5 Transporter Gene Expression and Association of Amino Acid Substitutions With Salt Tolerance Across Rice Genotypes
Source: Front Plant Sci. 2019 Nov 4;10:1420. doi: 10.3389/fpls.2019.01420 (PMC6843544; doi:10.3389/fpls.2019.01420)
Supplement: Supplementary file 10 [file Table_6.docx]

**Supplementary Table 6:** Model Validation score by different tools.

| Model | Template | identity | GMQE | Verify3d | ERRAT | Rampage |
| --- | --- | --- | --- | --- | --- | --- |
| **Model1** | 3pjz.1.A | 15.61 | 0.4 | 54.22 | 73.33 | 83.5 |
| **Model2** | 6hra.1.A | 14.92 | 0.39 | 44.18 | 76.9932 | 84.7 |
| **Model3** | 4j7c.1.I | 22.25 | 0.45 | 70.06 | 84.93 | 84.6 |
| **Model4** | 5but.1.E | 22.31 | 0.45 | 56.81% | 72.8195 | 83.4 |
| **Model5** | 5mrw.1.A | 14.92 | 0.41 | 54.22 | 67.7852 | 86.7 |
| **Model6** | 4j9u.1.A | 15.61 | 0.4 | 54.7 | 78.93 | 86.1 |
