# Supplementary material for: HKT1;5 Transporter Gene Expression and Association of Amino Acid Substitutions With Salt Tolerance Across Rice Genotypes
Source: Front Plant Sci. 2019 Nov 4;10:1420. doi: 10.3389/fpls.2019.01420 (PMC6843544; doi:10.3389/fpls.2019.01420)
Supplement: Supplementary file 11 [file Table_7.docx]

**Supplementary Table 7:** Distribution of different residues in different regions of the Ramachandran plot computed in the PROCHECK program

| Residue in most favoured region [A,B,L] | 363 | 80.5% |
| --- | --- | --- |
| Residue in additional allowed region [a,b,l,p] | 63 | 14.0% |
| Residue in generously allowed region[~a,~b,~l,~p] | 11 | 2.4% |
| Residue in generously allowed region | 14 | 3.1% |
| Number of non-glycine and non-proline residues | 451 | 100.0% |
| Number of end-residues (excl. Gly and Pro) | 2 |  |
| Number of glycine residues (shown as triangles) | 44 |  |
| Number of proline residues | 24 |  |
| Total number of residues | 521 |  |
